# A single-cell multi-omics assay for simultaneous measurement of vector copy number and protein expression in CAR T cells

**DOI:** 10.1016/j.omta.2026.201738

**Published:** 2026-04-15

**Authors:** Yilong Yang, Saurabh Parikh, Chieh-Yuan Li, Lindsey Murphy, Khushali Patel, Qawer Ayaz, Mahir Mohiuddin, John T. Elliott, Hua-Jun He, Zhiyong He, Samantha Maragh, Benjamin Schroeder, Terry J. Fry, Amanda Winters, Shu Wang

**Affiliations:** 1 Mission Bio, 300 Utah Avenue, Suite 210, South San Francisco, CA 94080, USA; 2 City of Hope, Duarte, CA 91010, USA; 3 Children’s Hospital Colorado, Aurora, CO 80045, USA; 4 National Institute of Standards and Technology, Gaithersburg, MD 20899, USA; 5 University of Colorado Anschutz, Aurora, CO 80045, USA

**Keywords:** vector transduction, vector copy number, single-cell, DNA sequencing, protein sequencing, VCN distribution, CAR T, cell therapy, gene therapy, lentiviral vector

## Abstract

Chimeric antigen receptor T cell (CAR T) immunotherapies have transformed cancer treatment. These therapies typically involve lentiviral vector-mediated CAR gene integration, followed by reinfusion into patients, necessitating rigorous quality control. Critical parameters, including transduction efficiency and vector copy number (VCN), must be accurately quantified. However, conventional gene transfer assays lack the resolution to capture cellular heterogeneity, relying on bulk population averages or labor-intensive clonal outgrowth methods. In order to address these limitations, we have developed an end-to-end solution from panel design to data analysis pipeline for targeted single-cell interrogation of transgene copy number. We applied this single-cell protein + DNA multi-omics VCN workflow to analyze a bi-cistronic CD19xCD22 CAR T product. Our VCN analysis exhibited high sensitivity, specificity, and average VCN linearity, as validated by an orthogonal method. Additionally, it uniquely revealed the unprecedented single-cell resolution of the VCN distribution profile. Furthermore, we quantitatively measured surface protein expression for lineage assignment, which revealed differential transduction percentages and VCN distribution patterns across cell lineages (e.g., CD4+ and CD8+ T cells), providing insights into factors potentially influencing treatment outcomes. This single-cell genomic/proteomic workflow could be a powerful research tool with potential clinical applications to enhance our ability to deliver optimal CAR T products to patients.

## Graphical abstract

**Figure:**
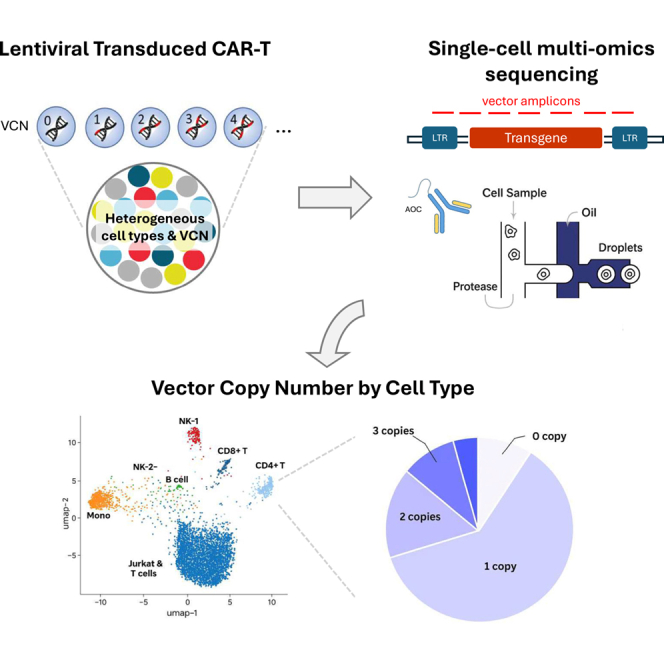

## Teaser

Yang and colleagues present a single-cell multi-omics platform enabling simultaneous analysis of vector genotype, copy number, and immunophenotype in CAR T cell products. This approach advances product characterization by revealing clonal heterogeneity and transgene variability, supporting safer, and more effective CAR T cell therapies.

## Introduction

Cell and gene therapies are revolutionizing the treatment of genetic disorders, including cancer and inherited diseases.^1^ Among the most transformative approaches are chimeric antigen receptor T cell (CAR T) immunotherapies, which leverage genetically engineered T cells to eliminate malignant cells expressing one or more target antigens.^2^^,^^3^^,^^4^^,^^5^^,^^6^ This therapy relies on introducing transgenes, most commonly through lentiviral transduction, to endow T cells with the desired specificity and functionality.^7^ To ensure the safety and efficacy of these modified cells, it is critical to accurately measure gene transfer efficiency and viral vector copy number (VCN) per cell. These parameters directly impact both the potency and potential adverse effects of the therapy.^8^ A high transduction rate with an optimal VCN enhances CAR T cell function, while excessive vector copies risk insertional mutagenesis and clonal expansion.^9^ Thus, balancing therapeutic efficacy and minimizing malignancy risks is crucial for CAR T therapy.

Conventional methods for quantifying VCN per transduced cell rely on the combination of multiple assays: e.g., fluorescence-activated cell sorting (FACS) analysis to determine transduction efficiency, and qPCR or droplet digital PCR (ddPCR) to determine sample VCN.^10^^,^^11^^,^^12^^,^^13^^,^^14^ These robust techniques are widely applicable but they have significant limitations. One major drawback is their inability to distinguish individual cells or identify clones with varying VCNs. For example, longitudinal studies of patients’ post-CAR T infusion have revealed heterogeneous clonal responses to tumors, emphasizing the need to identify the most therapeutically effective VCNs within specific clones.^15^^,^^16^^,^^17^ Moreover, high-resolution quantification is vital to understanding the heterogeneity of CAR T products. Current bulk analysis methods provide only average VCN values, which fail to account for the distribution of vector copies among individual cells. This is a critical limitation, as different VCN distributions, even with identical averages, can have significantly different clinical impacts. Indeed, fundamentally, an engineered cell is the therapeutic agent, and thus, addressing this shortcoming is pivotal for advancing CAR T therapy.

Previously, single-cell analysis for estimating VCN has been facilitated by Fluidigm’s microfluidic-based single-cell preparation platform integrated with ddPCR.^18^ However, this method is limited by low throughput, a complex operational process, and high result variability. Since the emergence of single-cell DNA sequencing technology enabled by Mission Bio’s Tapestri platform, several software packages with copy number (CN) detection capabilities have been developed, including Optima^19^ and KaryoType.^20^ However, these tools are primarily designed to detect chromosome-level ploidy changes by either comparing amplicon reads to diploid reference cells at genome-wide scale or to a reference gene. As a result, their resolution is limited, making them less suitable for analyzing customized vectors, which are much smaller than chromosomes. Additionally, the reproducibility and precision of CN distribution detection using these methods have not been fully validated. In this study, we present a high-throughput, microfluidic, and amplicon-based single-cell protein + DNA multi-omic workflow designed to quantify transduction efficiency, VCN distribution, as well as protein expression, across ∼10,000 cells simultaneously. We validated the performance of the single-cell VCN assay using clonal VCN cell lines with defined VCNs, confirmed by orthogonal methods, and included control cell lines transduced with different vectors.

We applied this single-cell protein + DNA multi-omic VCN workflow to analyze a bi-cistronic cluster of differentiation CD19 and CD22 CAR T product. Our VCN analysis confirmed the distribution of VCN in the product closely adhered to the expected Poisson distribution throughout the expansion, suggesting a low risk of biased functional clonal expansion favoring cells with high VCN pertaining to samples in this study. Additionally, besides interrogating vectors in the genome, using a 45-plex protein assay, we quantitatively measured surface protein expression of individual cells for lineage assignment. This revealed differential transduction percentages and VCN distribution patterns across cell lineages (e.g., CD4+ and CD8+ T cells), providing insights into factors potentially influencing treatment outcomes. This single-cell multi-omics transduction and VCN analysis package provides an all-in-one analytical solution that offers multiple functionalities, including transduction efficiency estimation, VCN distribution profiling, and average VCN calculation. By providing high-resolution insights into CAR T cell heterogeneity, this approach aims to enhance the precision of CAR T product characterization and improve clinical outcomes.

## Results

### Design of targeted DNA sequencing panel for VCN evaluation

Custom amplicon panels were designed to interrogate both human genome and vector sequences. For the human genome, 99 amplicons (human panel) were distributed across 22 chromosomes with 2–9 amplicons per chromosome, to serve as endogenous controls for comparative analysis between control and test samples. These amplicons were also utilized for demultiplexing different cell lines sequenced in the same run by exploiting unique genetic signatures. For vector sequence, two panels were created: one targeting the National Institute of Standards and Technology (NIST) control vector (vector panel 1) with 11 amplicons and another targeting the University of Colorado CD19xCD22 CAR T vector (vector panel 2) with 18 amplicons. The vector amplicons were designed to maximize the coverage of vector sequences. All designed amplicons were optimized for uniform coverage, with an amplicon size range of 183–270 bp. The combination of human panel and vector panel 1 was first tested on the NIST control cell lines for VCN model evaluation and then the combination of human panel and vector panel 2 was used for the CAR-expressing samples. Detailed information about amplicons included in each combined panel are reported in Table S1.

### Development of VCN caller model

**Figure 1:**
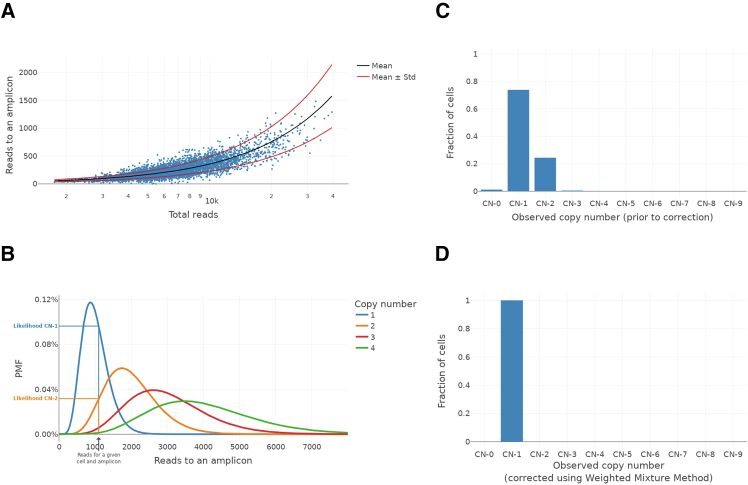
Development of the single-cell VCN caller model Example data from the NIST VCN = 1 control (run ID VCN35_V1) are shown to illustrate variability across amplicons and cells. While these figures display representative dataset, all NIST control cell lines (VCN = 0–4) were used collectively to develop and validate the bioinformatics pipeline (A) Distribution of reads from amplicon VCN_AMP619 plotted against the total reads within each Jurkat cell in a total of 4,377 cells, showing the variability across different total read depths. Black trend line: mean read counts modeled by the negative binomial (NB) distribution. Red lines: range defined by the mean ± standard deviation, showing that most cell reads fall within this interval. (B) Visualization of read count variability using the VCN_AMP619 amplicon (example). This figure displays the probability mass function (PMF) generated from the Negative Binomial (NB) distribution, demonstrating the observed cell-to-cell variability in sequencing read counts for cells with copy numbers (CNs) of 1, 2, 3, and 4. Arrow: the CN of the cell amplicon given the read counts on the x axis is the CN with maximum likelihood (blue), and the quality of the CN call is defined as its difference from the second most likely CN (orange). (C) Fraction of cells with copy number estimated by the NB distribution model of all vector amplicons for this run with only VCN = 1 NIST control cells. (D) Copy number distribution for the same run after correction using the weighted mixture method.

The development of the single-cell DNA VCN caller model involved addressing variations in amplicon performance and cell-to-cell signal consistency. To evaluate the variability in amplicon performance, we analyzed the distribution of read counts for individual amplicons across different total reads in a cell. Figure 1A illustrates the spread of read counts, highlighting differences in amplicon performance under varying sequencing depths. Additionally, cell-to-cell variation in CN profiles was observed, resulting in large variance of the read distributions for various CNs to overlap, necessitating robust correction methods (Figure 1B). Figures 1A–1D present representative examples from a single dataset and are not intended to depict the full range of variability observed across all experiments, although similar patterns were consistently observed in other datasets. To mitigate these challenges, an individual vector amplicon was modeled using a three parameter (const, x1, alpha) negative binomial (NB) distribution. “Const” is used to model amplicon performance variability. Amplicons with relatively lower reads are assigned lower const values while amplicons with relatively higher mean reads have higher const values. To model the total read depth dependence, “x1” is used. The higher this value, the stronger the dependence between the amplicon read counts and the total read counts. This is also larger for amplicons with relatively higher mean reads. “Alpha” is used to model the cell-to-cell variability. The larger this value, the larger the overlap between various CNs. Amplicons with relatively higher mean reads tend to have a lower alpha compared to amplicons with lower mean reads. This makes amplicons with a higher relative mean reads better predictors of CN in general. In addition, the algorithm’s statistical framework further mitigates technical bias. Specifically, the total read depth parameter (x1) and the cell-to-cell variability parameter (alpha) are designed to accommodate the characteristic signature of artifacts like doublets or multiplets (co-encapsulation leading to increased total read counts). This correction relies on the assumption that the control and test cell populations share a similar likelihood of doublet events, resulting in comparable variations in human amplicon reads. By integrating this shared variance profile, the fitted NB distribution effectively models and accounts for the potential systematic bias introduced by doublets or multiplets. Based on these three parameters, we got the expected value (mean) and the standard deviation (SD) for various total reads in a cell. As can be seen in the Figure 1A, this fit closely matches the observed cell read count distributions for that amplicon.

After computing the negative binomial distribution (NBD) parameters that best fit the read counts of cell and vector amplicons with known CNs from control cell line data, these parameters were extrapolated to predict distributions for other CNs. The likelihood of a given cell-amplicon read count belonging to a specific CN was then calculated based on the estimated distributions. The CN of that cell-amplicon is the CN corresponding to the NBD for which the likelihood (or log likelihood) is the maximum (Figure 1B). The difference between the log likelihood of the most likely and the second most likely CN is the quality of the call. The larger the difference, the higher confidence of the call. The log-likelihoods for each CN are summed across all amplicons within a cell, and the final CN for the amplicon group is assigned to each cell based on the highest total log likelihood. The CN quality for these is also defined in the same way, as the difference between the highest and second highest log-likelihoods.

To reduce background noise from cell-to-cell variability and improve accuracy of CN proportion estimates, we developed the weighted mixture method (WMM). This algorithm generates simulated datasets for various CNs that incorporate the empirically observed correlations between closely tiled amplicons, ensuring that systematic effects such as polymerase run-on are represented in both control and test data. Using these simulations, we iteratively adjusted the proportions of expected CN states to minimize the distance between the observed and expected CN distributions, yielding the optimal CN proportion estimates for each sample.

Following this approach, the predictions of the proportion of various VCNs in the samples (Figure 1D) are clearly closer to the fraction of cells we expect in the run for all CNs compared to the original distribution (Figure 1C) with fall-off clones (cell clones exhibiting VCNs that differ from the predominant CN(s) exist in the cell population.), enabling accurate reconstruction of VCN profiles.

### Assessment of transduction efficiency and VCN caller performance

To determine the performance of measuring transduction efficiency—that is, how many cells in a sample harbor vector(s) in the genome—19 single-cell runs were performed using mixtures of vector-positive clonal Jurkat cell lines (VCN 1, 2, 3, and 4) and vector-negative Raji cells, with 2–3 replicates for each group (Table S2 samples VCN35-VCN57). All runs demonstrated good panel uniformity (≥81%) with a median per-run coverage of 58× (median of the run-specific average read counts per cell per amplicon, Table S3). Raji and Jurkat cell lines were identified by comparing the genotype in the human genome amplicons to the in-house database of known germline variants for these cell lines. Then VCN estimation for all the identified Jurkat and Raji cells (test cells) was performed for each run using the Jurkat cells in the run VCN39_V4 as control with predefined VCN 4. Each cell’s transduction status was characterized based on the presence (VCN >0) or absence (VCN = 0) of vector amplicons in that cell. Based on a total of 82,103 Jurkat and Raji cells, the single-cell percentage transduction efficiency assay had 99.6% specificity and 99.9% sensitivity (Table S4). The high transduction specificity indicates a very low rate of artifactual VCN-positive calls among negative control cells (VCN 0), consistent with a low frequency of barcode hopping.

**Figure 2:**
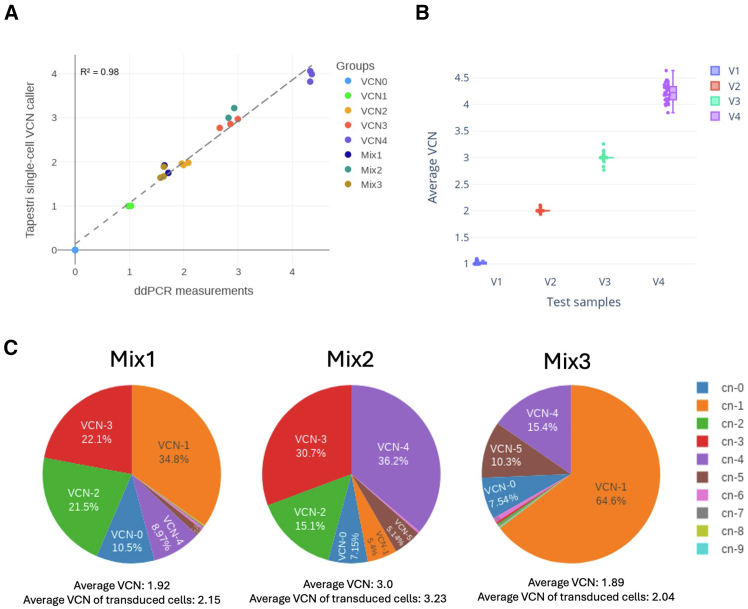
Assay qualification of single-cell VCN model using NIST VCN control cell lines (A) Correlation of average VCN estimated by Tapestri single-cell VCN caller and the orthogonal ddPCR measurements across runs with different NIST VCN reference cell lines, including mixtures with predefined ratios. Each point represents an individual Tapestri run, color-coded by groups of cell line compositions. The dashed line represents the linear regression fit; R^2^ value was determined using Pearson’s correlation coefficient. (B) Distribution of average VCN estimated by all VCN controls (VCN 1–4) for each test sample. The boxplot shows the median average VCN (horizontal line), interquartile range (box), and data spread (whiskers). Each data point represents a pairing of a test sample replicate with a control sample replicate. Each VCN test sample consists of three replicates, each of which was analyzed using all control cell lines with VCN values ranging from 1 to 4. (C) Distribution of single-cell VCN across three Tapestri runs with different NIST reference cell line compositions. Each pie chart represents the proportion of cells with different VCN within a given run. Samples with similar average VCN (Mix 1 and Mix 3) have different VCN distribution. Details about runs containing mixtures (Mix#) were provided in Table S2. VCN pie charts of all mixed runs are shown in Figure S2.

Assay qualification using conventional ddPCR was restricted to bulk-average VCN measurements because ddPCR cannot resolve single-cell heterogeneity. Mean VCN values estimated by the Tapestri VCN caller were compared with ddPCR results from the same samples to assess linearity across a range of seven distinct expected population averages consisting of reference control cell lines and mixtures (Table S5; Figure 2A). The average SD of replicates measured by Tapestri (mean SD = 0.08) demonstrated comparable precision to ddPCR method (mean SD = 0.05). Linear regression comparing ddPCR and Tapestri average VCN values across the full validated range (0–4) demonstrated strong proportionality (slope = 0.93, 95% confidence interval [CI]: 0.87–0.99; R^2^ = 0.98 Figure 2A). This linearity was further confirmed by residual analysis (r ≈ 0, *p* ≈ 1), establishing an unbiased quantitative response and confirming the absence of signal saturation or loss of precision at the upper end of the tested range.

To assess the algorithm’s robustness in average VCN estimation using different control cell lines as references, we performed the assay on samples containing fixed NIST control cells with CNs ranging from 1 to 4, each in triplicates. Using each run with different VCN as reference to determine the other runs’ average VCN, the results show low variation, with coefficient of variation (CV) of 2.76%, 1.62%, 2.48%, and 4.04%, respectively for VCN controls from VCN 1 to VCN 4. (Figure 2B). This suggested that no significant bias was introduced for the prior VCN test using VCN39_V4 as control cells.

For VCN distribution, the proportion of cells in categories from VCN 0 to ≤ VCN 9 was shown in Table S5 and Figure S1. For the 12 Tapestri runs with a single Jurkat VCN reference cell line, on average 93% of cells were called as the exact VCN value as expected for the corresponding reference cell lines, suggesting high precision on VCN estimation.

However, outlier VCN calls, particularly those with unexpectedly high or low values, necessitated an in-depth re-evaluation to rule out technical artifacts, such as doublets (or multiplets) involving the co-encapsulation of more than one cell per droplet. Our VCN measurement strategy is robust against doublets formed by cells with identical VCNs, as the vector-to-genome ratio remains unchanged. Conversely, when VCNs differ, the resulting doublet produces an artificial VCN equal to the arithmetic average of the two true VCNs. This averaging consistently results in a VCN call that is lower than the maximum VCN of the parent cells, manifesting as false-positive data points in the intermediate CN range. To quantify this artifact, we examined the proportion of outlier VCNs across two quality control scenarios: single VCN control runs (*n* = 12), where we quantified cells with VCNs lower than the expected target; and Mix 3 runs (*n* = 3), where we quantified cells with VCNs between the lowest (VCN 1) and highest (VCN 4) expected calls. The proportion of cells with VCN values lower than expected across these runs averaged 5% (Table S5), which is consistent with the anticipated low doublet frequency. Furthermore, the highest proportion of these low VCN calls was observed in the run containing the VCN 3 cells, which aligns with our independent ddPCR measurement of VCN = 2.66 (also lower than the expected integer VCN call).

Conversely, no technical artifact is known to predictably cause unexpectedly higher VCN calls. The proportion of cells with VCN values exceeding the expected level across all Tapestri runs averaged only 2%. This low frequency suggests the high outliers are unlikely due to a major systematic error. Notably, even when using the maximum VCN 4 as a reference, a small fraction of cells was detected with VCN >4 in eight runs (e.g., 6% in run VCN57_Mix3). This observation is consistent with bulk ddPCR measurements performed on the same materials, which reported mean VCNs ranging from 4.32 to 4.35 across three replicates. This evidence suggests that these higher than expected VCN calls reflect true biological variation—specifically, an average VCN slightly above the nominal integer value of the cell line, rather than a technical sequencing artifact.

To further assess the nature of these outlier populations, we performed a comparative analysis of normalized read counts between cells called with the expected VCN and those flagged as lower or higher than expected VCN. For each experimental run, we compared the absolute read differences for both the human amplicon reads (genomic control) and the vector amplicon reads (VCN signal). The results (Table S6) showed that these outlier cells had insignificant differences (*p* > 0.99) in human amplicon reads compared to the cells with the expected VCN. This finding strongly indicates that differences in total input genomic DNA, which would be the signature of a technical artifact like an unequal doublet or significant background noise, are not the primary cause of the VCN deviation. Instead, the low VCN cells consistently exhibited significantly lower (*p* < 0.001) vector amplicon reads, and the high VCN cells consistently exhibited significantly higher (*p* < 0.001) vector amplicon reads, relative to the expected VCN population. This pattern directly supports the conclusion that the outlier VCN calls accurately reflect the cells’ true low or high VCN signals, rather than stemming from confounding technical issues like doublets or sequencing bias.

The pie charts in Figure 2C show the VCN distribution calculated for one replicate of each of the three mixture groups (see Figure S2 for other replicates). “Mix 1” was purposefully mixed with VCN 1 as the dominant population and VCN 4 the least. On the contrary, “Mix 2” was purposefully mixed with VCN 4 as the predominant population and VCN 1 the least. “Mix 3” was mixed to demonstrate the ability to detect a bi-modal distribution of VCN, where the sample contains VCN 1 and VCN 4 samples. For all mixtures, we spiked-in 10% of vector-negative Raji cells for VCN 0. The single-cell VCN pipeline was able to determine the distribution of the VCN profile accurately as expected. Specifically, while “Mix 1” and “Mix 3” both have similar sample-wide average VCNs of 1.92 and 1.89, respectively, the VCN distributions were drastically different (“Mix 1” VCN 1–4’s respective proportions = 34.8%, 21.5%, 22.1%, and 8.97%; “Mix 3” VCN 1–4’s respective proportions = 64.6%, 0.3%, 0.4%, and 15.4%). This highlights the resolution of the single-cell VCN assay informing of potential efficacy differences in cell and gene therapy (CGT) products due to the distribution profile, whereas average bulk VCN were not able to differentiate. Furthermore, since the single-cell assay directly interrogates the vector transduced into each cell to measure the percentage transduction and calculate sample average VCN, it can also provide the value of average VCN per transduced cell in one assay (Figure 2C, labels below pie chart).

Lastly, besides counting the vectors in a cell, due to the single-cell VCN assay’s next-generation sequencing (NGS) single-nucleotide resolution readout, the pipeline can also survey the vector integrity in terms of insertion-deletion (indels) or single nucleotide variant (SNV) in the vector sequence where amplicons were designed. To illustrate this, in all the 19 runs containing Jurkat reference cell lines, we identified a spontaneous mutation occurring at vector position 408:G/A in six of those Tapestri runs and they all contain a mixture of multiple VCN reference cells. Strong correlation (Pearson’s correlation *p* value: 0.006, *n* = 6) were observed between the fraction of mutated cells and the fraction of VCN = 4 cells (Table S7) in the mixture. No mutated cell detected in one of the two Mix 1 replicates (VCN54_Mix1) likely due to the lowest proportion (1.7%) of VCN 4 cells. This mutation was also absent in all the non-mix replicates containing a single VCN 4 reference cell line. As these two negative samples originated from a separate cell processing batch, processed on different dates, the discrepancy is likely attributable to biological variation present in the initial cell preparations (batch effect). We acknowledge this variability and consider it a factor for further investigation in future studies focused on mutation stability. Since VCN reference cell lines have identical germline genotypes, to characterize the vector mutation profile within VCN 4 cells without the possible interference of other closer VCN reference cells being mistakenly called VCN 4, we selected three Mix 3 replicates containing mixtures of only VCN 1 and VCN 4 cells for further analysis. The median variant allele frequency (VAF) of vector mutation in VCN 4 Jurkat cells were 22.3%, 23.1%, and 23.5% for the three replicates, respectively, suggesting the occurrence of mutation in one out of four copies. A combined heatmap of genotype and amplicon normalized read counts were shown for each replicate in Figure S3.

### CD19xCD22 bi-cistronic vector transduction and VCN assay qualification

We applied the single-cell transduction and VCN workflow to a CGT and biologically relevant vector, where we designed an amplicon panel tiling across a CD19 and CD22 bi-cistronic vector 11,^14^ to confirm that our single-cell VCN assay linearity performance was not vector-sequence dependent. For this CAR vector, we established the assay performance baseline using Jurkat-derived clonal cell lines with VCN 0 and VCN 2. VCN 2 was selected because it reflects the typical average VCN of 1–2 reported for clinical CAR T cell products,^12^ and was the only stable transduced clone obtained, as higher VCN proved toxic to cells and are difficult to clonally expand. Ten test runs were performed with mixtures of these cell lines at different ratios (VCN 0:VCN 2 = 100:0, 50:50, 25:75, and 0:100), with three replicates at each level. VCN estimation was benchmarked against an additional run (MUR62) that included the same VCN 2 clonal cell line as a predefined control (VCN = 2; Table S2). For transduction percentage analysis, the assay demonstrated excellent linearity (R^2^ = 0.998) with coefficients of variation (CV) across replicates ranging from 0.4% to 1.3% (Table S8). Furthermore, we analyzed the 100% VCN 0 (all cells expected to be vector negative) and 100% VCN 2 (all cells expected to be vector positive) runs, as well as the vector-negative GM12878 cells, and assigned each cell’s transduction status based on the presence or absence of vector amplicons in that cell. From a total of 30,285 cells, we calculated the percentage transduction assay for the CAR vector has a sensitivity of 99.2% and specificity of 98.8% (Table S9).

**Figure 3:**
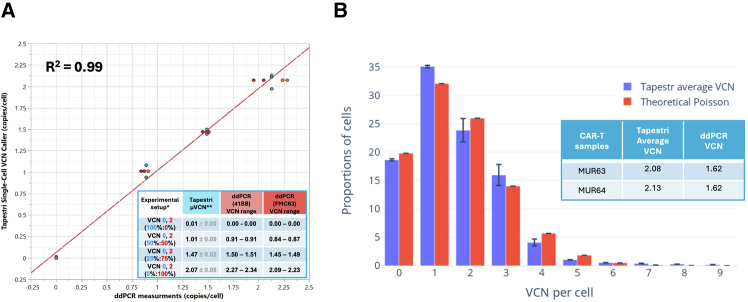
Assay qualification of single-cell VCN model using CD19xCD22 bi-cistronic CAR transduced cells (A) X-Y scatterplot of the average VCN for each sample called using Tapestri VCN caller vs. ddPCR measurement (average of two separated ddPCR probe assays targeting 41BB and FMC63 region) performed on the same admixture samples. The red line represents the linear regression fit; R^2^ value was determined using Pearson’s correlation coefficient. The average and range of replicate measurements are shown in the bottom right table. ∗Mixtures percentage based on empirical cell counting; ∗∗Units in copies/cell; standard deviation based on 3 technical replicates for Tapestri runs. (B) Comparison of Tapestri single-cell VCN caller measured, and Poisson model predicted cell proportions across vector copy numbers in CD19xCD22 transduced primary T cells. The bar graph displays the proportions of cells with specific vector copy numbers as determined by the Tapestri single-cell VCN caller (blue bars) from two replicate Tapestri runs and as predicted by the theoretical Poisson distribution model (red bars). Error bars represent the SD from replicate runs. The table shows the average VCN measured by single-cell Tapestri data and ddPCR data.

To assess average VCN linearity, we analyzed average VCN for the admixtures at four dilution levels with defined VCN ratios (Table S8). These values were then compared to results from two orthogonal ddPCR assays targeting the 41BB and FMC63 elements of the vector.^14^ The analysis demonstrated strong concordance between the single-cell VCN values and those obtained from the ddPCR assays, with a linearity R^2^ value of 0.99 (Figure 3A). Regression results obtained using CAR-Jurkat cells were concordant with those from the NIST reference cell line (consistent slope and proportionality), confirming that the assay accurately measures VCN across different vectors and is free of detectable vector-specific bias in VCN estimation.

A noteworthy observation is that for replicates of 100% VCN 2 samples, the average VCN measured by ddPCR assay 41BB was 2.27–2.34, and ddPCR assay FMC63 measured as 2.09–2.23. The discrepancy of average VCN measured by the two separate ddPCR assays may be due to template/assay-specific performance characteristics and variations in the degree of optimization. For the single-cell VCN distribution, for the three runs with a single VCN 2 control cell line, on average 86% of cells were detected as exact VCN 2, and on average 7% of cells have VCN >2.

### CAR T product VCN distribution

After validating the panel and VCN workflow, the single-cell VCN caller was applied to analyze lentiviral-transduced CAR T products in primary T cells. A developmental batch of CAR T cells was cultured for 7 days post-transduction and analyzed using the single-cell workflow (run IDs MUR63-64 Table S10), and the average VCN per transduced cell were determined to be 1.62 and 1.99 copies, respectively using ddPCR. By using the Jurkat VCN 2 cells in Tapestri run MUR62 as control, the average VCN was estimated to be 2.08–2.13, and the single-cell VCN distribution revealed that 81.5% of the sample was vector-positive, with VCN 1 being the dominant population, representing approximately 35%. Specifically, due to the lentiviral transduction procedure, each cell has an independent probability of being transduced by a viral particle, we would expect the VCN distribution follows a Poisson distribution 11.^10^ Indeed, for the CAR T product, the overall VCN distribution is closely aligned with the theoretical Poisson distribution (μ = 1.62) with a correlation of R^2^ = 0.98 (Figure 3B). This suggests the manufacturing process for this batch of cells did not introduce significant VCN-dependent clonal expansion within the time frame. Notably, while the average VCN was 1.62, there are still portions of cells (∼2%) estimated to harbor five or more vector copies (Table S10). Further longitudinal studies for VCN dependent gain of function assays could investigate whether cells with higher VCN counts exhibit clonal hematopoiesis phenotypes.

### Lineage-specific VCN analysis in CAR T

The single-cell workflow enables simultaneous measurement of surface protein expression and VCN analysis, facilitating cell-lineage-specific VCN distribution insights (see materials and methods). This is accomplished by pre-staining cells with antibody-barcode conjugates, followed by processing through the standard single-cell DNA analysis workflow. The antibody-specific barcodes are incorporated into the NGS readout alongside DNA amplicon data, with cell-specific barcodes linking genotypic and phenotypic information at the single-cell level. The protein panel consists of 45 unique cell surface antigens (including principal lineage antigens). The DNA panel included the CD19xCD22 CAR VCN panel (vector panel 2 that was validated in the previous section that identifies transduction percentage, VCN distribution) with a 99-plex human DNA panel that is used to determine unique SNVs in the genome (see Table S1 for panel details).

**Figure 4:**
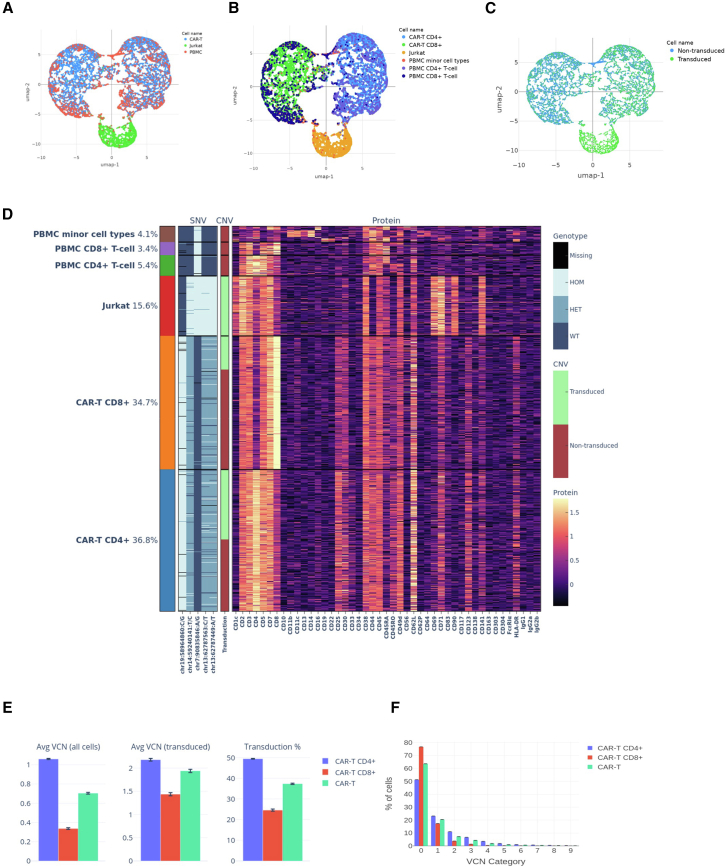
Visualization of cell clustering and genotype, immunophenotyping, and vector transduction status for one of the three replicate Tapestri runs with CAR T cell product (run ID: MUR65) (A) UMAP projection of clustered cells, colored by spiked-in cell lines. (B) The same UMAP projection with cells colored by cell type determined by immunophenotyping. (C) UMAP projection with cells colored based on vector transduction status. (D) Heatmap displaying germline variant genotypes, transduction status, and antibody marker expression profile across all cells. The compositional breakdown of PBMC minor cell types: monocyte: 1.75%, B cell: 0.93%, NK cell: 1.02%, CD69+ T cell: 0.37%. UMAP and heatmap plots for the other replicate Tapestri runs are provided in Figures S5 and S6. (E) VCN statistics across all three replicate runs with CAR T product cells, showing VCN profiles in the total CAR T cells (green), CD4+ CAR− T cells (blue) and CD8+ CAR T cells (red). Error bars represent the SD from replicate runs. (F) Proportion of cells with each VCN across three T cell types in all replicates.

The experiment was performed in triplicate, each run with the CAR T sample of interest spiked-in with VCN 2 control cell lines (Jurkat) as vector positive reference, as well as peripheral blood mononuclear cells (PBMCs) (separate donor, vector null) as vector negative reference and cell surface marker positive reference. In this experimental setup, cells are clustered using uniform manifold approximation and projection (UMAP) based on surface protein expression (Figures 4A–4C; Figures S4 and S5), with major cell lineages identified (e.g., B cells, CD4+ and CD8+ T cells, NK cells, etc.; Figure 4B). Notably, VCN 2 reference cells with a Jurkat background exhibit a distinct protein expression profile despite originating from the T cell lineage (Figure 4A). The accompanying heatmap (Figure 4D) highlights the SNVs used to differentiate among vector-positive Jurkat cells, vector-negative PBMCs, and CAR T cells. This dataset also showcases the potential utility of the protein + DNA workflow in CGT contexts, enabling the clustering of cells by surface markers, distinguishing potential donor samples (Jurkat cells) from recipient samples (PBMCs) through SNV profiling, and analyzing vector status distributions accordingly (e.g., vector-positive Jurkat vs. vector-negative PBMCs as shown in Figure 4C).

Building on the Tapestri runs described earlier, we applied the protein + DNA multi-omics VCN analysis on CAR T products using the spiked-in VCN 2 cell line as control. From the transduction percentage analysis, as expected, Jurkat cells are vector positive (>99.09%) and PBMCs are vector negative (<0.09%), whereas the CAR T cells have a ∼37% transduction efficiency. Furthermore, different T cell lineages were identified based on CD4+ and CD8+ expression and their VCN distributions and transduction percentage were measured using the VCN pipeline. Based on the analysis, albeit the CD8+ T cell to CD4+ T cell ratio ranges from 0.94 to 0.98, the VCN distribution profile (Figures 4F; Table S5) is different and their average VCN value is ∼0.34 and ∼1.06, respectively (∼1:3 ratio; Figure 4E). Significant differences were observed between CD4+ and CD8+ T cells in average VCN across all cells (*t*_3_ = 68.62, *p* < 0.001), average VCN within the transduced subset (*t*_3_ = 16.44, *p* < 0.001), and overall transduction percentage (*t*_3_ = 46.54, *p* < 0.001). However, when comparing single-cell VCN distributions, no statistically significant differences were detected between the two lineages by permutation testing (*p* = 0.095). This finding indicates that despite the similar distribution shape, the significant differences in VCN averages and transduction percentages are rooted in a systematic upward shift in the magnitude of vector integration in CD4+ T cell, reflecting both improved efficiency and a bias toward higher VCNs within the transduced subset. These results highlight the potential of our single-cell platform to characterize lineage-specific integration patterns that are inherently masked by bulk analysis. Although the study was not powered to draw broad biological conclusions, the data serves as a proof of concept for how single-cell resolution distinguishes between global transduction trends and the stochastic expansion of high-copy clones, which is a critical distinction for identifying the root causes of VCN variability.

To illustrate the utility of protein expression data in addressing important questions, for example in the context of CAR T cell exhaustion, we defined an exhaustion-like phenotype (effector memory with concurrent CD69 and HLA-DR expression) using a proxy definition based on the markers available in the current antibody panel (CD69, HLA-DR, CD25, CD45RO, and CD62L).^21^ This rare exhaustion-like phenotype was variable between CAR+ (∼11%–13%) and CAR-cell populations (∼8%–15%) (Table S11). Only one replicate showed statistically significant enrichment in CAR+ cells (Fisher exact *p* = 0.03), while the others were non-significant or trended opposite. These data suggest that in these particular CAR T cells after *in vitro* expansion, while CAR vector positive cells may drive a small subset of cells toward a proxy-defined exhaustion-compatible state, the overall CAR+ population does not. For future studies, additional contextual antibodies, such as canonical markers of T cell exhaustion (e.g., PD-1), could be included to expand the antibody panel.

## Discussion

Traditional methods for assessing VCN and transduction efficiency, such as qPCR and ddPCR, offer population-level data that fail to capture the cell to cell variability critical for understanding clonal dynamics and detailed makeup of a CAR T cell product. Single-cell resolution data are especially valuable to the field of cell and gene therapies, where a modified cell is the basic biological functional unit for therapeutic purposes, while poorly characterized modified single cells with unintended edits can cause potential adverse transformation and clonal expansion.

The Food and Drug Administration (FDA) guidance on CAR T product release recommends flow cytometry to determine the percentage of CAR-positive cells, as well as molecular methods to determine VCN.^22^ Recent clarification of the guidance notes that average VCN should be expressed relative to percentage of CAR-positive cells, as using the denominator of total cells can underestimate the vector integration rate. This current good manufacturing practice (GMP)/good clinical practice (GCP) workflow requires multiple modalities to assess the CAR T product—both *in vitro* and *in vivo*—and yet only provides an estimate of the true single-cell composition.

In this study, we demonstrated multi-omics, single-cell quantification of transduction efficiency, VCN distribution, population average VCN, and average VCN per transduced cells, and immunophenotyping in one single assay. The single-cell level VCN analysis provided unprecedented resolution and insights to assess the potential functional efficacy and safety for CAR T therapy, as well as a way to better understand the biology of these therapies. This work represents an assay qualification performed within a single-laboratory setting, designed to establish key analytical performance characteristics linearity, specificity, and sensitivity using defined control cell lines. Because conventional approaches such as ddPCR cannot resolve cell-to-cell variability, the validation against these methods necessarily focused on bulk-average VCN values. The unique value of the single-cell VCN assay lies in its ability to capture distributional heterogeneity and integrate VCN with protein expression at the single-cell level capabilities beyond the reach of bulk methods and highly informative for product characterization. As such, this single-cell multi-omics platform has the potential to accelerate and streamline both process development and release testing of engineered cell therapies.

For percentage transduction measurement, the single-cell DNA sequencing method demonstrated high sensitivity (99.2%–99.9%), specificity (98.8%–99.6%), and assay linearity (R^2^ = 1) using cell lines transduced with different vectors. The single-cell assay also provides percentage CV consistent with existing approaches, demonstrating its reliability. This is largely attributed to the single-cell transduction assay’s utilization of multiple vector amplicons (*n* = 11–18 amplicons used in this study) to directly determine the presence or absence of the vector per cell via NGS readout, analyzing thousands of cells in parallel.

Beyond detecting the presence (VCN >0) or absence (VCN = 0) of the vector in each cell using vector amplicon reads, we developed a single-cell VCN pipeline that determines the average VCN and VCN distribution of the sample. The results showed excellent concordance with orthogonal ddPCR measurements (R^2^ = 0.98–0.99). Additionally, the average VCN per transduced cell, a critical quality attribute (CQA) for CAR T products, is provided. Compared to conventional methods combining FACS for transduction percentage (based on CAR expression, not all integrated vectors have expression) and qPCR or ddPCR for average VCN, this single assay offers a straightforward method to characterize CAR products by directly analyzing the vectors integrated into the genome. Furthermore, the NGS readout enables single nucleotide resolution SNV analysis within the regions covered by the designed amplicons, potentially allowing simultaneous monitoring of vector integrity and instability, including truncations and mutations.

The unique capability that the single-cell VCN assay provides is the insight into the distribution of VCN of the sample, as opposed to providing a bulk average. As demonstrated in the mixture study with VCN control cell lines (Figure 2C), two samples with the similar average VCN value can have drastically different VCN distributions that directly contribute to the efficacy of the product. Consequently, the utility of this assay in a manufacturing context lies in its capacity for profound process characterization and optimization. While these high-resolution insights provide invaluable visibility into cellular heterogeneity, we recognize that such data must be carefully contextualized. Rather than serving as an immediate lot-release criterion for therapies with established clinical records, this single-cell workflow functions as an advanced analytical tool that empowers developers to achieve deeper process understanding and tighter manufacturing controls. By resolving the actual cellular composition, rather than relying on potentially deceptive population averages, manufacturers can proactively optimize upstream processes to minimize batch-to-batch variability and study underlying product dynamics. Furthermore, resolving these distributions provides a critical platform for investigating clonal outgrowth and insertional mutagenesis, offering the necessary resolution to support future longitudinal studies from initial manufacturing through post-transplantation. For example, in CAR T products expanded for 7 days in culture, the observed VCN distribution closely resembled the expected theoretical Poisson distribution, suggesting no significant functional clonal outgrowth during this manufacturing time frame. Future longitudinal studies could further characterize VCN distribution profiles at various stages of CAR T manufacturing and treatment.

To further contextualize the advantages of this approach, we compared it to a previously published ddPCR-based single-cell VCN method,^18^ which normalizes vector copies against diploid reference genes to derive VCN estimates via Bayesian modeling. Both the methods can detect transduced (VCN >0) and non-transduced (VCN = 0) cells, but Santeramo’s approach achieves ∼80% accuracy relative to bulk in average VCN and analyzes only ∼70–400 cells per assay, limiting its scalability. The NGS-based method here, leveraging the Mission Bio Tapestri platform, amplifies 11–18 vector amplicons across >7,000 cells on average per run (Table S3), achieving ∼94% concordance with ddPCR bulk VCN (Table S5) and detecting rare high-VCN events (>5 copies) at 0.1% frequency (Table S5). Unlike ddPCR’s diploid normalization, this approach calibrates against control cell lines with known VCN (e.g., 1–4), improving accuracy for high-VCN predictions, where diploid assumptions may be confounded. Both the methods can be adapted to different CAR constructs by primer or amplicon redesign, but this workflow uniquely integrates multi-omic profiling (DNA + protein) to link VCN with CAR expression or mutations. A limitation of this approach is its higher cost and computational complexity relative to ddPCR, though these are offset by its scalability, sensitivity, and added biological resolution.

This high-throughput, multiplexed approach seamlessly integrates with the multi-omics workflow, combining protein expression analysis with DNA-based VCN quantification for lineage-specific insights. For instance, differential transduction efficiency and VCN distributions observed in CD4+ versus CD8+ T cells underscore how such heterogeneity might influence therapeutic efficacy. CD8+ T cells, as the primary cytotoxic subset, could benefit from optimized VCN distributions to maximize functional activity while mitigating risks associated with high transgene expression.^23^^,^^24^^,^^25^ Beyond these primary classifications, further differentiation into subsets such as stem cell memory T cells (T_SCM_), central memory T cells (T_CM_), and effector memory T cells (T_EM_) imparts distinct functional attributes to the CAR T products.^26^^,^^27^ This comprehensive antibody marker panel facilitates the precise phenotypic identification of specific T cell subsets, enabling a detailed assessment of their distinct contributions to the potency and persistence of the CAR T product. This enhanced resolution represents a key strength of our platform, providing insights into lineage-specific patterns that can inform optimizations in CAR T manufacturing and ultimately enhance both therapeutic efficacy and safety.

In addition, the multiplex capabilities of the targeted amplicon-based single-cell method enable the simultaneous analysis of multiple transgenes or vector elements as well as other regions of the genome of interest. Notably, when combined with gene-editing analysis of indels, this approach allows for the examination of co-occurrences between edits and vector insertions at single-cell resolution. These features are especially valuable for the development and characterization of next-generation CAR designs that incorporate multiple functional components.^28^^,^^29^

While this study establishes a robust foundation for the technical advancement, there are several areas for future improvement. One notable limitation is the reliance on VCN reference control cell lines for VCN quantification. To address this, we propose future modifications to the methodology, incorporating the use of spike-in plasmids as an alternative calibration strategy. Another strategy would be to design and validate amplicons that target conserved elements common to various vectors, including the modular components of conventional CAR structures,^30^ and provide these as cataloged amplicon panels. These adjustments would not only simplify the workflow but also enhance the method’s scalability and reproducibility across different laboratories. Secondly, to provide a technical and algorithmic proof of concept for this novel methodology, this assay employed VCN 1–4 controls, with VCN 2 included specifically for CAR T sample analysis. The selected VCN control samples represent a dynamic range consistent with average VCNs frequently reported for CAR T products.^12^ Inclusion of higher VCN controls could further refine calibration and expand dynamic range, enabling more robust linearity assessment at elevated integration levels. This may be particularly useful in research contexts where VCNs above the clinical threshold are investigated. Additionally, despite the strengths of this approach, the current bioinformatics workflow requires further integration and automation. At present, the VCN estimation and other computational components operate as separate analytical steps, which may limit accessibility and ease of use for a broader research and clinical community. To address this, we are actively working on developing an automated pipeline that seamlessly integrates these components into a unified platform. This advancement will enhance usability, streamline data processing, and make this methodology more practical and widely applicable for researchers and clinicians seeking efficient and scalable CAR T cell analysis solutions.

In conclusion, the single-cell protein + DNA multi-omic VCN workflow represents a transformative tool for advancing CAR T product characterization. By providing high-resolution insights into transduction efficiency, VCN distribution, lineage-specific transduction patterns, and vector integrity, this platform has the potential to refine CAR T manufacturing and quality control processes. Ultimately, such advancements may contribute to improving the precision, safety, and efficacy of CAR T therapies, paving the way for better patient outcomes.

## Materials and methods

### VCN control cell lines

Parental Jurkat cell line (Clone E6-1, ATCC, Manassas, VA, USA) and four clonal transduced Jurkat cell lines with 1, 2, 3, or 4 copies of lentiviral vector(s) (kindly provided to NIST by Lentigen Gaithersburg, MD) were cultured in RPMI-1640 growth medium (ATCC, Manassas, VA, USA) supplemented with 10% heat-inactivated fetal bovine serum (FBS, Thermo Fisher Scientific, Grand Island, NY, USA) and 2 mM Glutamax (Thermo Fisher Scientific, Grand Island, NY, USA). The relative VCN was determined through qPCR and ddPCR, functional integration of the vector was detected through NGS, and differential expression of GFPs was measured by flow cytometry for each VCN clonal cell line. Consensus values of VCN and integration sites in these VCN reference cell lines were achieved in a recent NIST organized interlaboratory study for VCN measurements.^31^ Each clonal cell line was fixed with Carnoy’s solution (3:1 volume of methanol and acetic acid) according to Mission Bio Tapestri Platform V2 – preparation of methanol fix cells for cell storage and Tapestri runs protocol and stored in 4°C. The clonal VCN cells were cultured and fixed in the NIST laboratory before being shipped to Mission Bio for analysis. Each clonal cell line was fixed with Carnoy’s fixative and stored in 4°C until use. The fixed cells were buffer exchanged by 1% BSA (Thermo Fisher Scientific, Carlsbad, CA, USA) solution in dulbecco's phosphate-buffered saline (DPBS) prior to running the single-cell experiments following Tapestri v.2 DNA user guide (Mission Bio, SSF, CA, USA).

### CAR T reference cells

Jurkat cells were utilized to establish clonal cell lines expressing the bi-cistronic CD19xCD22 dual CAR vector. Cells were thawed and plated at an initial density of 1.0 × 10^6^ cells per 1.25 mL. Transduction was performed using the lentivirus encoding the CAR construct at a range of multiplicities of infection (MOI) from 180 to 720, with a separate well included for the mock-transduced control. Following a 24 h incubation, cells were harvested, counted, and resuspended at a final density of 1.0 × 10^5^ cells/mL in R10 media (RPMI supplemented with 10% FBS and 1× GlutaMAX). The cells were maintained in culture for 1 week, with media replenished every 3 days. For clonal expansion, cells were then stained for CD45 and viability (using an Annexin V/AAD panel) and single-cell sorted into 96-well plates. Transduced Jurkat clonal cell lines were validated by droplet digital (ddPCR) measurement using methods previously described. 11^14^ Briefly, bulk transduced Jurkat cells were single cell sorted into 96-well plates using a FACSAria cell sorter and then cultured to generate clonal populations. The VCNs of these clonal populations were measured using two separate CAR-specific ddPCR assays targeting 41BB and FMC63 regions of the bicistronic CAR with albumin as a reference gene. Clonal populations with VCN of 0 and 2 were used in this study as known VCN references. Samples containing a mixture of control reference transduced with CAR T vectors were processed for single-cell sequencing using Mission Bio’s Tapestri platform in triplicates. For single-cell multi-omics analysis, PBMC and VCN 2 samples were stained with a 45-plex oligo conjugated antibodies (total seq-D, heme oncology panel, BioLegend) followed by Tapestri workflow.

### CAR T cell samples

Both the CAR-expressing primary T cells and the CAR T cell product were derived from a normal, healthy donor leukopak and generated as process development materials for the bicistronic CD19xCD22 CAR construct. T cells were isolated using Miltenyi anti-CD4 and anti-CD8 magnetic beads; the CD4:CD8 ratio was not controlled. The CAR T cell product was manufactured at the Gates Biomanufacturing Facility (Aurora, CO, USA) under process development conditions using a G-Rex100 vessel with TexMACS medium supplemented with 3% human blood group AB (AB) serum and 12.5 ng/mL each of IL-7 and IL-15 on the CliniMACS Prodigy platform (Miltenyi Biotec, Germany). Leukopaks were purchased through approved vendors. no further institutional ethical approval was required for this study.^14^ Selected T cells were seeded on day 0, transduced on day 1 with the University of Colorado CD19xCD22 vector construct at an MOI of 30, and harvested on day 8 following culture and expansion. CAR T cells were cryopreserved in CS10 (BioLife Solutions). Transduction efficiency was confirmed via flow cytometry. Cells (10,000 per test) were stained with a viability dye (Fixable Dye e780, Thermo Fisher Scientific) and a panel of markers, including CD19-Fc, CD22-Fc, and a lyophilized 6-color T cell cocktail (BioLegend). Fluorescent minus one control ensured accurate gating. Annexin V staining was used for viability assessment, and data were acquired on a Gallios flow cytometer (Beckman Coulter) and analyzed with Kaluza software. We conducted multiple sequencing runs using the cell lines described earlier. The details of each sequencing run, including the cell line compositions, are summarized in Table S2. Each run was designed to evaluate the VCN caller’s performance under different cellular compositions, ensuring a comprehensive assessment of detection accuracy across varying conditions.

### Amplicon panels design

To generate data used for the development of the VCN estimation pipeline, we designed a vector amplicon panel (vector panel #1) containing 11 amplicons (ranging 183–270 bp insert size) using Tapestri Designer.^32^ The amplicons span across the vector and cover all major elements of the vector sequence. For the subsequent investigation of CAR T vectors across multiple sample types, including a Jurkat-derived CAR-transduced cell line, CAR-transduced primary T cells, and CAR-transduced healthy donor CAR T cells, the second vector amplicon panel (vector panel #2) containing 18 amplicons were designed following similar design criteria. Among those, 11 amplicons were used to cover 61% of CAR bases, and 7 amplicons were designed to cover the vector sequence on both sides of CAR. All vector amplicons were designed using the default parameters of the panel design pipeline, which automatically determines the optimal tiling of amplicons across the full vector sequence to ensure maximum coverage.

The human panel was designed to be combined with the VCN panels and used for cell identification and normalization purposes. It was built using the hg19 reference genome and a predefined list of targets. The initial target list comprised 675 SNPs with allele frequencies between 0.4 and 0.6, based on the snp147 common SNP dataset from University of California, Santa Cruz (UCSC) genome browser, as well as 321 genomic regions present in single or multiple copies in the human reference genome to account for CN variations relevant for robust VCN modeling. To minimize sequence variation at primer binding sites while maximizing variability within the amplicon inserts for distinguishing germline variants between samples, additional primer selection criteria were applied on top of the default Tapestri designer pipeline settings. These additional filters excluded primers containing SNPs, primers with SNPs within 1–2 nucleotides downstream of the primer’s 3′ end, and primers with SNPs within 15 nucleotides upstream of the 5′ end of the forward primer, to avoid interference with the Tapestri barcode adapter. Furthermore, each selected amplicon was required to contain at least one SNP within its insert region. An initial set of 519 amplicons was designed and tested in internal unpublished screening experiments (S.W., data not shown). From this set, a subset of 99 amplicons with mean read counts greater than zero was randomly selected to comprise the final human amplicon panel, which covers 22 chromosomes (excluding chromosome X and Y) with 2–9 amplicons per chromosome.

### Digital PCR assays

The digital PCR assay used for identifying sample average VCN follows the user guide from QX200 Droplet Digital PCR System (Bio-Rad, CA, USA). In brief, the sample was processed using DNeasy Blood and Tissue Kits for DNA Isolation (QIAGEN) and quantified by nano-drop. DNA was mixed with ddPCR master mix and respective assays targeting vector elements for quantification and an assay annealing temperature of 55°C.

For reference VCN vectors:

RRE forward primer: AAACTCATTTGCACCACTGC.

RRE reverse primer: AATTTCTCTGTCCCACTCCATC.

RRE probe: (FAM) TGTGCCTTGGAATGCTAGTTGGAGT.

RPL32 forward primer: CAAGGAAAGACGAGCTGTAGG.

RPL32 reverse primer: GGGCAGTTGCATCTTCATATTC.

RPL32 probe: hexachloro-fluorescein (HEX) AGCTGCAGGCAGAAATTCTGGTAGT.

For CAR T vectors:

Albumin forward primer: CAAGCCCTGAAGCTCAACTC.

Albumin reverse primer: GATTTGTGTGGGCATGACAG.

Albumin MGB probe: (VIC) AGTTGCTGTCATCTC.

41BB/CD3ζ forward primer: GAGACCCGTGCAGACAACC.

41BB/CD3ζ reverse primer: TCTTCTGCCGAGGTTCAGC.

41BB/CD3ζ MGB probe: (FAM) CGCTTATCAACAGGGC.

Furin/FMC63 forward primer: TCTGGTACCCCCGATCCT.

Furin/FMC63 reverse primer: GCTTGTGGTCTGGGTCATCT.

Furin/FMC63 MGB probe: (FAM) TGCTCAAGCAGGCTG.

### Single-cell DNA and protein sequencing

Mission Bio’s Tapestri platform enables simultaneous analysis of genomic regions of interest and the quantification of surface protein expression across 10,000–20,000 of cells by encapsulating and barcoding individual cells, followed by bulk NGS (Figure S6). In brief, the single-cell sequencing workflow involves a two-stage microfluidics process comprising cell encapsulation, barcoding, multiplex emulsion PCR, and NGS library preparation. For the DNA and protein workflow, cells are first stained with 45 unique primary lineage antibody-oligo conjugates (Total Seq-D Heme Oncology Cocktail, BioLegend). These oligo tags serve as unique identifiers that quantitatively capture cell surface protein expression.

During cell encapsulation, individual cells are rapidly isolated into nanoliter droplets containing lysis buffers, ensuring that each droplet contains material from a single cell. These droplets are incubated at 50°C to release DNA from chromatin, a critical step to enable uniform single-cell genomic analysis. Following cell lysis, barcoding droplets are formed by merging the lysates with cell barcode beads and PCR reagents. This barcode acts as a molecular tag that permanently links all DNA and protein molecules back to their cell of origin. PCR amplification then occurs within these barcoded droplets, which physically separate single-cell reactions and prevent cross-cell mixing, and allow the amplification targeting specific genomic regions within single cells. Subsequently, the emulsions are broken, and the amplified DNA is pooled for standard NGS library preparation. Sequencing was conducted using a 2 × 150 bp paired-end format on the Illumina NextSeq 550 platform. Comprehensive details on utilizing Tapestri’s single-cell sequencing, including necessary materials, equipment, and protocols, are available in Mission Bio’s Tapestri v.3 User Guide (or v.2) for DNA-only workflows and the Tapestri DNA and Protein v.3 User Guide for combined workflows.

### Single-cell DNA and protein data analysis

The analysis of Mission Bio Tapestri single-cell DNA and protein sequencing data follows a standardized workflow. First, the Tapestri DNA/DNA + Protein Analysis Pipeline (v.3.0) is used for read alignment, cell calling, and the generation of h5 files containing processed sequencing data. Cell calling was performed using only the human amplicons in the panel. Tapestri data quality metrics including sequencing and panel performance were reported in Table S3. DNA germline variant genotyping is then performed for sample demultiplexing to assign cells to their respective cell lines according to an in-house curated database. Cells of different genetic backgrounds within the sample (e.g., Jurkat/Raji cell lines) were identified when >60% of their known germline variants matched the reference database. Because most Tapestri runs contained multiple cell lines or samples except for three runs using the Jurkat VCN 0 cell line, this approach effectively excluded the majority of doublets or multi-cell clusters involving cells with distinct genotypes. Doublets formed by cells with identical genetic backgrounds were further evaluated in the following VCN analysis by identifying instances where the observed CN differs from the expected value.

The number of cells identified per sample is summarized in Table S3, and all identified cells were included in subsequent VCN estimation. For downstream data analysis and visualization, all h5 files were read into the Mosaic v.3.5 python package.^33^ Amplicon read counts are normalized by cnv.normalize_reads function. Protein data are normalized on a per-sample basis using the NSP (noise-corrected and scaled protein counts) protocol using the protein.normalize_reads function within Mosaic. Cell types are assigned using the protein assay clustering and relabeling (PACE) module in Mosaic by applying the default antibody marker expression profile for standard PBMC cell types. UMAP cell clustering and visualization are performed following the published Mosaic workflow, with modified parameters including three principal-component analysis (PCA) components and 50 UMAP neighbors. Finally, heatmaps are generated to visualize the integrated DNA, copy number variation (CNV), and protein data, displaying single-cell genotypic and phenotypic profiles.

To assess potential CAR T cell exhaustion, we utilized available protein expression markers from the single-cell dataset. From the three replicates of Tapestri DNA and protein datasets, CAR T cells identified by DNA genotype and excluding cells with sticky protein markers (expression of any of the three antibody markers: IgG1, IgG2a, and IgG2b ) were selected. We defined a proxy exhaustion-like phenotype as effector memory cells (CD45RO + CD62L−) co-expressing CD69 and HLA-DR. Marker expression was binarized using a positivity threshold of normalized protein reads as 0.7. Cells were then stratified by CAR status (CAR+ if VCN >0; CAR-if VCN = 0). Frequencies of the exhaustion-like phenotype were then compared between CAR+ and CAR− populations using Fisher’s exact test, and percentages and odds ratios were reported.

### VCN estimate algorithm

#### Individual amplicon modeling

For vector amplicons, targets with an average read depth ≤5 were excluded from VCN modeling to avoid low-confidence estimates. All human genomic amplicons were retained without filtering. An individual vector amplicon with average reads is modeled using a three parameter (const, x1, alpha) NB distribution. Since the read counts to an amplicon are dependent on the total reads of the cell, const and x1 were used to model the mean of the read counts, where log (mean) = const + x1× log (total reads of each cell). Since the variance is proportional to the square of mean, alpha was used to model the variance of each VCN of a vector amplicon in a square form of the NB distribution where,

*variance* = *mean* + *alpha* × *mean*ˆ2.

Alpha: alpha was estimated from the NB parameter for the VCN used for NB modeling. For other VCNs, the NB parameter alpha is extrapolated by polynomial fitting with the average amplicon reads of all the amplicons and scaled by predicted VCN.

Mean: average amplicon reads estimated by the const and x1 parameters from the previous step were then scaled by predicted VCN.

#### Copy number predicted for amplicon and cell

Using the average reads and variance estimated earlier, the likelihood of each vector amplicon corresponding to each predicted VCN was calculated using an NB model. The CN of that cell-amplicon is the CN corresponding to the NB distribution for which the likelihood is the maximum. The difference between the log likelihood of the most likely and the second most likely CN is the CN quality of the call. Similarly, the most likely CN and CN call quality for a group of vector amplicons for a cell was determined.

#### Weighted mixture method

Finally, the proportion of cells with various CNs in a run was determined using a WMM to compare the proportion of various CNs estimated from original cell/amplicon reads (observed) vs. simulated and correlated reads (expected) described earlier. This is done by minimizing the SAD (sum of absolute difference) between the observed distribution and the expected distribution by varying the proportions (a.k.a weights) of the expected CNs.

Specifically, the first step was to simulate amplicon read counts independently for each CN state by sampling from a NBD parameterized from control data, ensuring that the total reads per cell matched those observed in the control run. Next, we quantified correlations of deviations from the mean read counts for all pairs of vector amplicons using the same control data. To incorporate this correlation structure into the simulations, we applied Cholesky decomposition to the correlation matrix and used it to transform the independent simulated values into correlated ones generating simulated datasets that reflect both the variability of individual amplicons and their experimentally observed correlations. The observed CN distribution was then compared to the simulated distributions across all CN states. The algorithm iteratively adjusted the weights assigned to each CN state to minimize SAD between the observed and expected distributions. After a maximum of 1,000 iterations or when SAD is ≤ 1/(10× total number of cells), the weight of observed and expected CN were determined and used to calculate the final proportion of cells with various CNs in a run.

#### Software execution for VCN analysis

For each Tapestri run, the data in the h5 files were first processed through the Mosaic package to generate data frames with aligned read counts for all cells and amplicons in the panel. These read counts data along with cell line control information were input into the CNVcaller v.1.0.1 pipeline (developed in-house), which executed the VCN caller algorithm. For the Tapestri runs containing VCN control cell lines that are genetically distinguishable from the test cells, the read counts from the spiked-in control cells were used as control reads input to model the amplicons. Otherwise, the external read control of NIST VCN 4 cells from external run VCN39_V4 were used. For a comprehensive list of Tapestri runs, refer to Table S2, which also provides parameter details on the VCN caller used for each run. This includes information on the source of control cells, the known CN of control cells, the human, and vector amplicons excluded, and the maximum CN allowed. All computations were performed in a Python-based environment, and batch processing was implemented for efficiency.

### Assessment of VCN assay linearity and statistical calculations

In addition to the primary analyses conducted using Mosaic and the VCN caller, various statistical calculations and custom analyses were performed using Python scripts. Correlation analysis, including the calculation of R-squared values, was conducted using the numpy and scipy.stats libraries. Chi-square tests for goodness of fit and independence were performed with Python packages scipy.stats.chisquare and scipy.stats.chi2_contingency, respectively. CV and SDs were computed using numpy.std and numpy.mean. The probability mass function (PMF) of the Poisson distribution was evaluated using scipy.stats.poisson.pmf to assess data distribution patterns.

To assess whether VCN calls flagged as outliers (lower or higher than expected VCN) were technical artifacts, we normalized read counts per cell to the total read depth and normalized reads per amplicon to its median using the cnv.normalize_reads function within the Mosaic package. Subsequently, we used one-tailed *t* tests to compare the read distributions of both the human reference and vector amplicon between each outlier group and the expected VCN cell population.

Linearity was assessed in two stages. First, we evaluated assay linearity using prototype VCN reference cells^34^ and engineered mixtures spanning a range of seven distinct expected population averages. We assessed the assay’s ability to accurately recover the expected average VCN from these blended testing materials and reported regression parameters and goodness of fit are reported in Figure 2A. Second, to test for potential vector-specific effects, the VCN caller was run on CAR T clonal samples at four dilution levels. The CAR T regression served as a confirmatory check and was compared to the NIST regression to assess agreement. Linearity was evaluated using a standard least-squares linear regression without weighting or transformations. A first-degree polynomial model was fitted to the observed VCN values using the Python numpy.polyfit function, and the coefficient of determination (R^2^) was obtained via Python package sklearn.metrics.r2_score. Proportionality was assessed by examining the (95% CI) of the slope and the R^2^ value. To detect potential curvature or systematic bias, a quadratic term was tested, and the correlation between residuals and fitted values was analyzed. All scripts were executed in a controlled computational environment, ensuring reproducibility and consistency across analyses.

## Data and code availability

Single-cell DNA sequencing FASTQ (text-based sequence format with quality scores) files of the samples used in this study, as well as h5 files generated through the Tapestri DNA + protein pipeline were deposited in NCBI under accession number NCBI: PRJNA1259689. Any additional data are available from the corresponding authors upon reasonable request.

## Acknowledgments

The authors extend their gratitude to the members of the Mission Bio team, NIST, and members of Winters and Fry labs for their insightful feedback on the manuscript and for their invaluable contributions through enlightening discussions. Certain commercial equipment, instruments, or materials (or suppliers or software) are identified in this paper to foster understanding. Such identification does not imply recommendation or endorsement by the National Institute of Standards and Technology, nor does it imply that the materials or equipment identified are necessarily the best available for the purpose.

A.W. received support from the 10.13039/100000002National Institutes of Health/National Institute of Child Health and Development 1K08CA279762-01, the 10.13039/100012869Morgan Adams Foundation, and Swim Across America. L.M. received support from the 10.13039/100000002National Institutes of Health under Ruth L. Kirschstein National Research Service Award T32CA236734 and Swim Across America. T.J.F. received support from the 10.13039/100006108National Center for Advancing Translational Sciences Colorado 10.13039/100006975CTSI grant number UL1TR002535 and the 10.13039/100000054National Cancer Institute U01C232486-01.

## Author contributions

A.W., C.-Y.L., and S.W. conceived and supervised the project; L.M., S.P., C.-Y.L., K.P., B.S., H.-J.H., Z.H., J.T.E., M.M., S.M., S.W., T.J.F., and A.W. contributed to the study design; L.M., AW., C.-Y.L., H.-J.H., Z.H., M.M., K.P. and Q.A. prepared the samples and performed the experiments; S.P. and Y.Y. developed the computational VCN pipeline and analyzed the data; L.M., Y.Y., S.P., C.-Y.L. and A.W. wrote the manuscript with contributions from all authors. All authors read and approved the final manuscript.

## Declaration of interests

Authors Y.Y., S.P., K.P., Q.A., B.S., S.W., and C.-Y.L. were employees of Mission Bio.

## Declaration of generative AI and AI-assisted technologies in the writing process

During the preparation of this work, the authors used available AI tools (e.g., ChatGPT) in order to enhance the quality of our writing. After using these tools, the authors reviewed and edited the content as needed and took full responsibility for the content of the publication.

## Supplemental information

Document S1. Figures S1–S6 and Tables S2, S4, S5, S7, S8–S11

Table S1. Amplicon panel used for this study

Table S3. Sequencing and panel performance metrics of all Tapestri runs used in this study

Table S6. Normalized read count differences between outlier and expected VCN populations

Document S2. Article plus supplemental information
