# Supplementary material for: A single-cell multi-omics assay for simultaneous measurement of vector copy number and protein expression in CAR T cells
Source: Mol Ther Adv. 2026 Apr 15;34(2):201738. doi: 10.1016/j.omta.2026.201738 (PMC13175768; doi:10.1016/j.omta.2026.201738)
Supplement: Document S1. Figures S1–S6 and Tables S2, S4, S5, S7, S8–S11 [file mmc1.pdf]

OMTA, Volume *34*

Supplemental information

A single-cell multi-omics assay for simultaneous measurement of vector copy number and protein expression in CAR T cells

Yilong Yang, Saurabh Parikh, Chieh-Yuan Li, Lindsey Murphy, Khushali Patel, Qawer Ayaz, Mahir Mohiuddin, John T. Elliott, Hua-Jun He, Zhiyong He, Samantha Maragh, Benjamin Schroeder, Terry J. Fry, Amanda Winters, and Shu Wang

Supplemental material

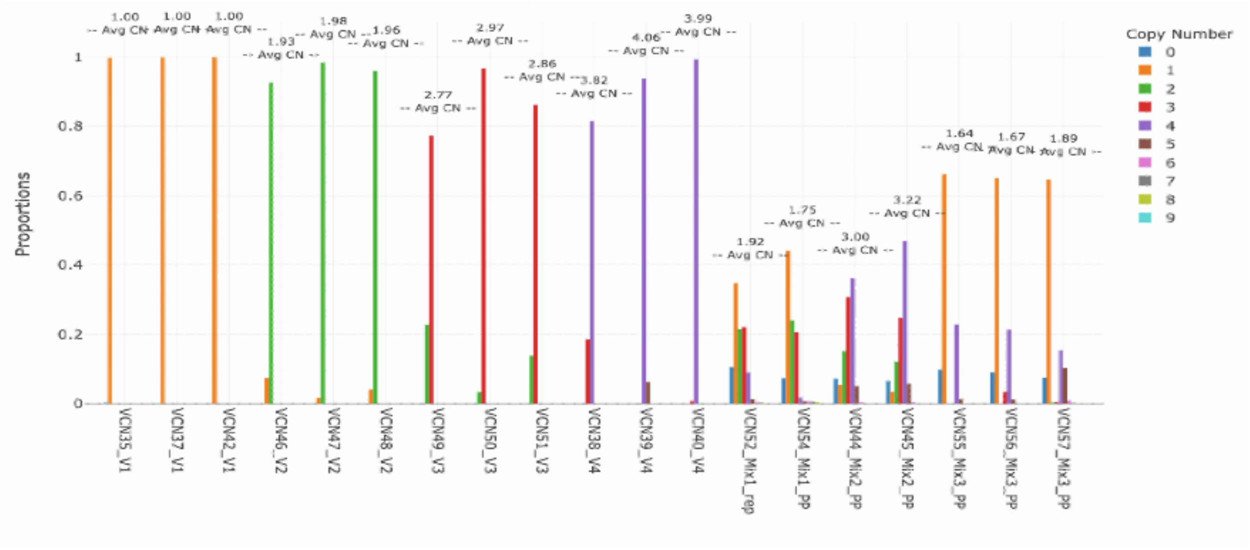

Figure S1, VCN distribution in NIST reference cell lines and mixture runs. Average VCN was shown at the top of each run.

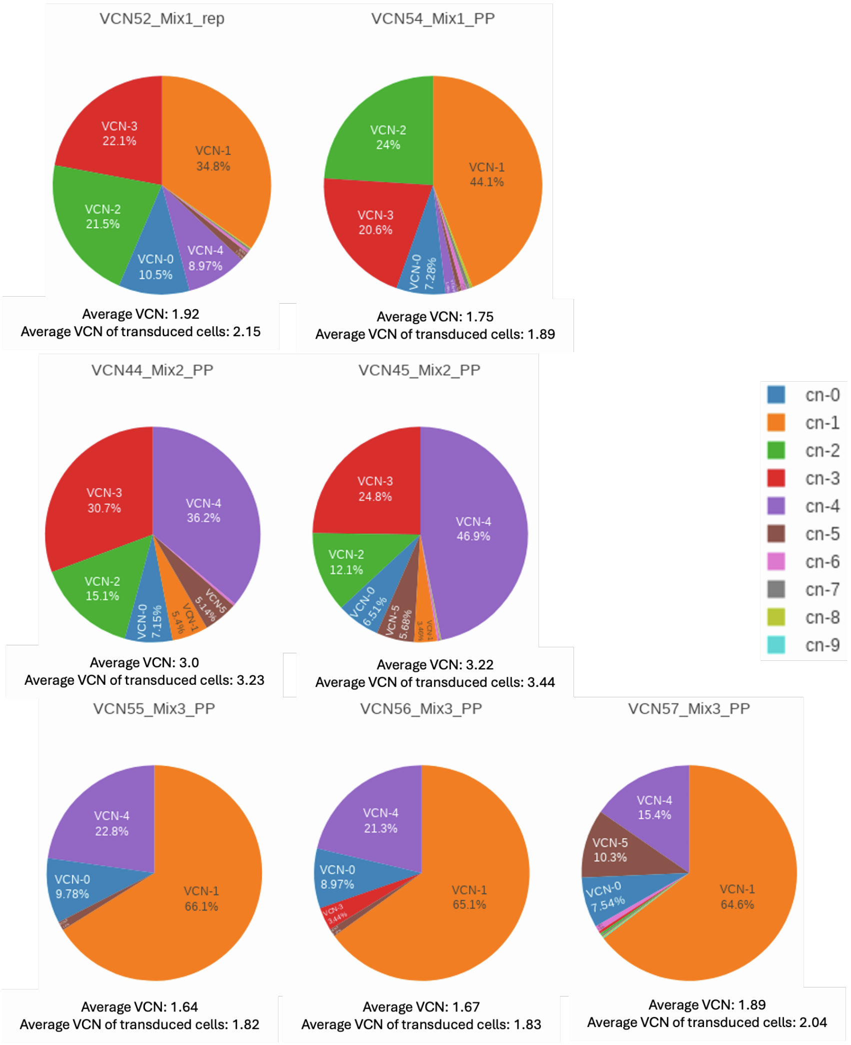

Figure S2. Distribution of single-cell VCN across three Tapestri runs with different NIST control cell line compositions.

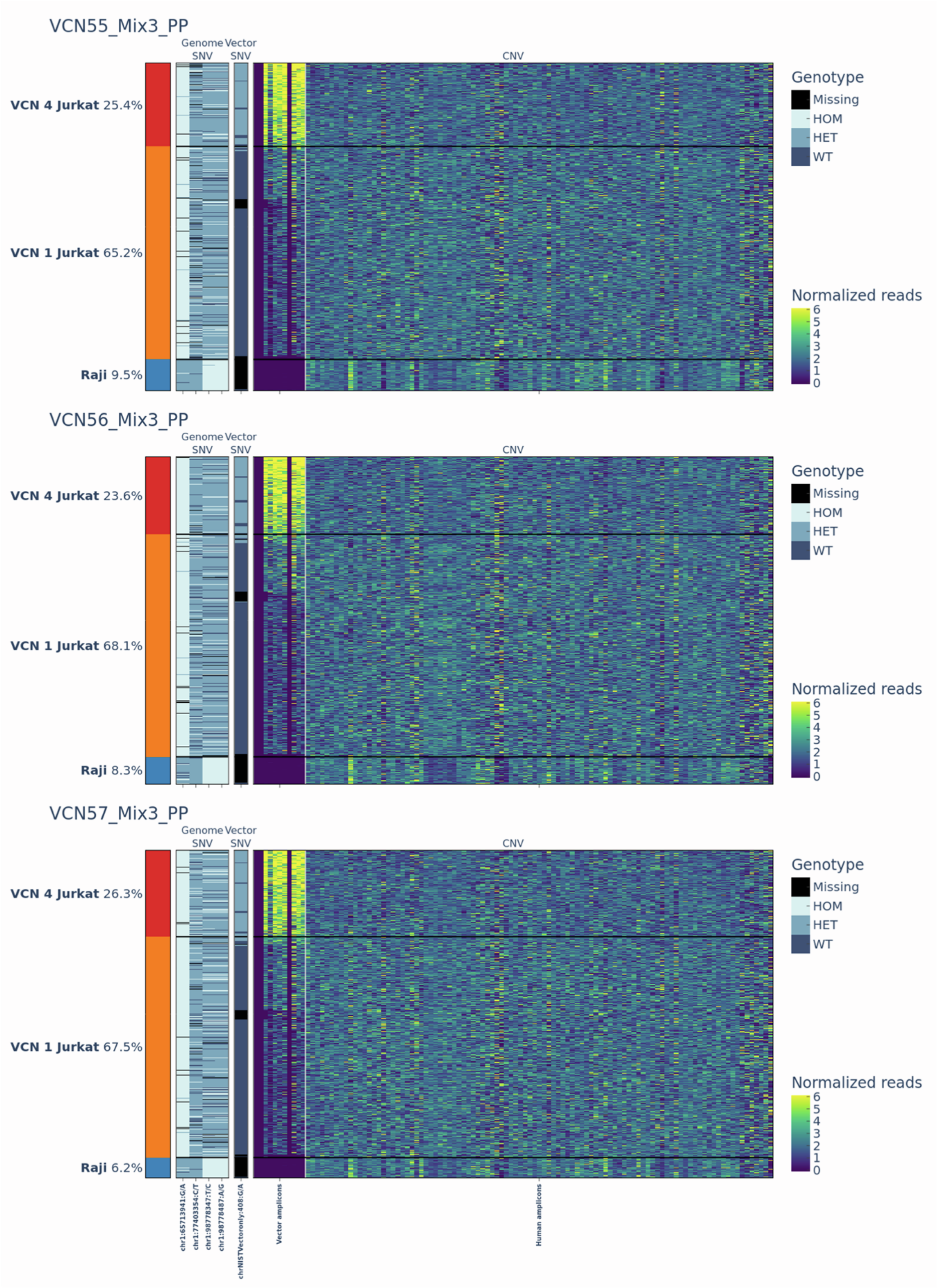

Figure S3. Single-cell SNV and CNV results of “Mix3” replicate runs.

“Genome SNV” selected human genome SNVs used to differentiate Jurkat and Raji cell lines. “Vector SNV” : G/A mutation at NIST vector sequence position 408 that were found only in VCN4 cells; “CNV” normalized read counts of all vector (first 11 amplicons on the left side) and human amplicons (the rest amplicons). Each row represents a cell. Cells were first clustered by different human genome genotype then by the normalized read counts of all vector amplicons. Since “Mix3” only contains NIST control cell lines of VCN 1 and VCN 4, Jurkat cell cluster with median VCN < 4 was named “VCN 1 Jurkat”, and Jurkat cell cluster with median VCN >= 4 was labeled as “VCN 4 Jurkat”. Data show that VCN1 and VCN4 cells exhibit uniform genomic profiles, with consistent single-nucleotide variant (SNV) and copy number variation (CNV) patterns across human amplicons. The primary differences between these cell populations lie in the vector-specific SNV genotypes and vector copy number (VCN). Although a small subset of VCN1 cells lacks complete vector SNV genotype data, the dominant vector SNV genotypes remain clearly distinct between the VCN1 and VCN4 populations.

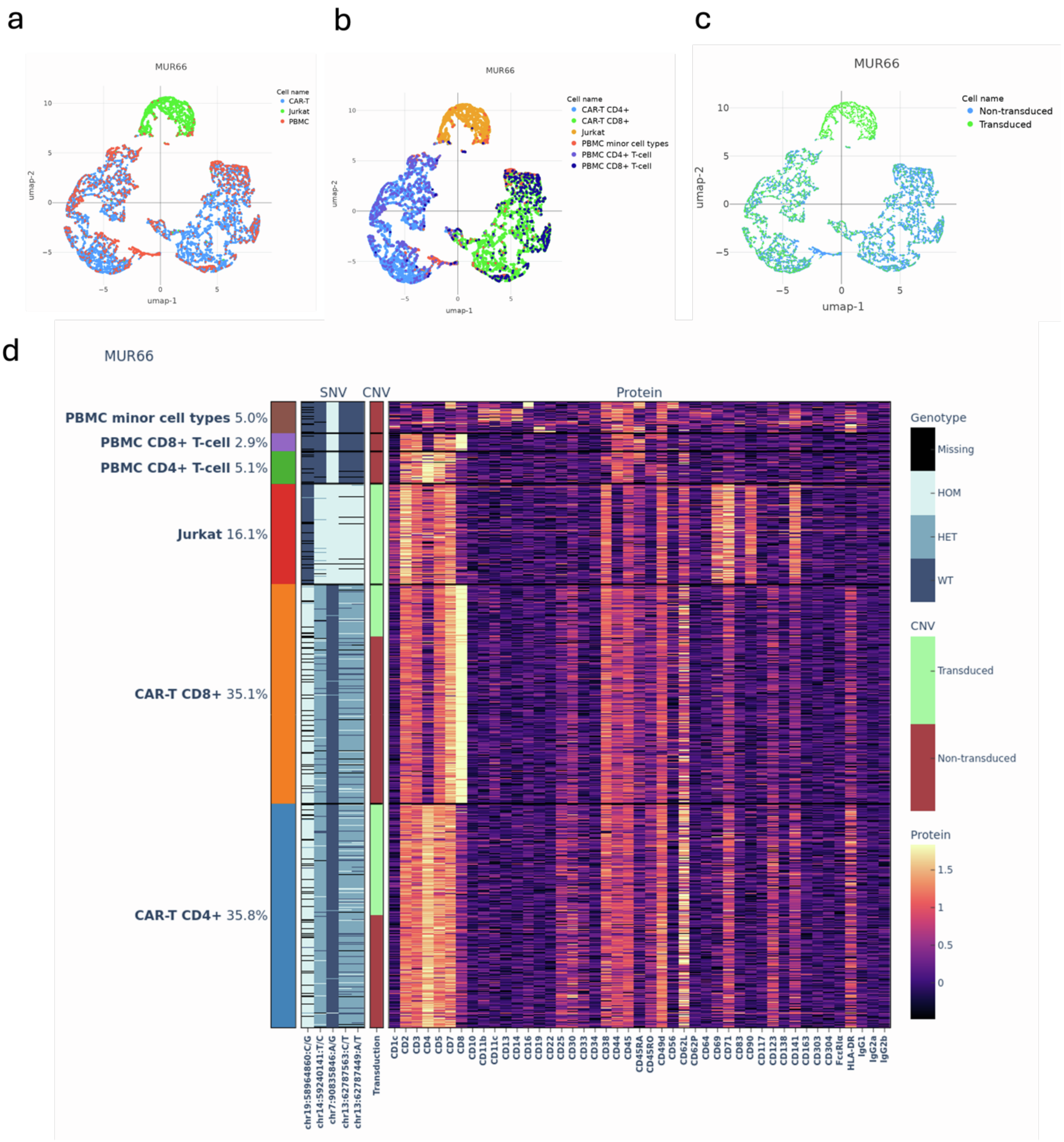

Figure S4. Visualization of cell clustering and genotype, immunophenotyping, and vector transduction status for one of the three replicate Tapestri runs with CAR-T cell product (MUR66). For interpretations please refer to Figure 4. The compositional breakdown of PBMC minor cell types: monocyte: 2.00%, B cell: 1.44%, NK cell: 1.00%, CD69+ T cell: 0.53%.

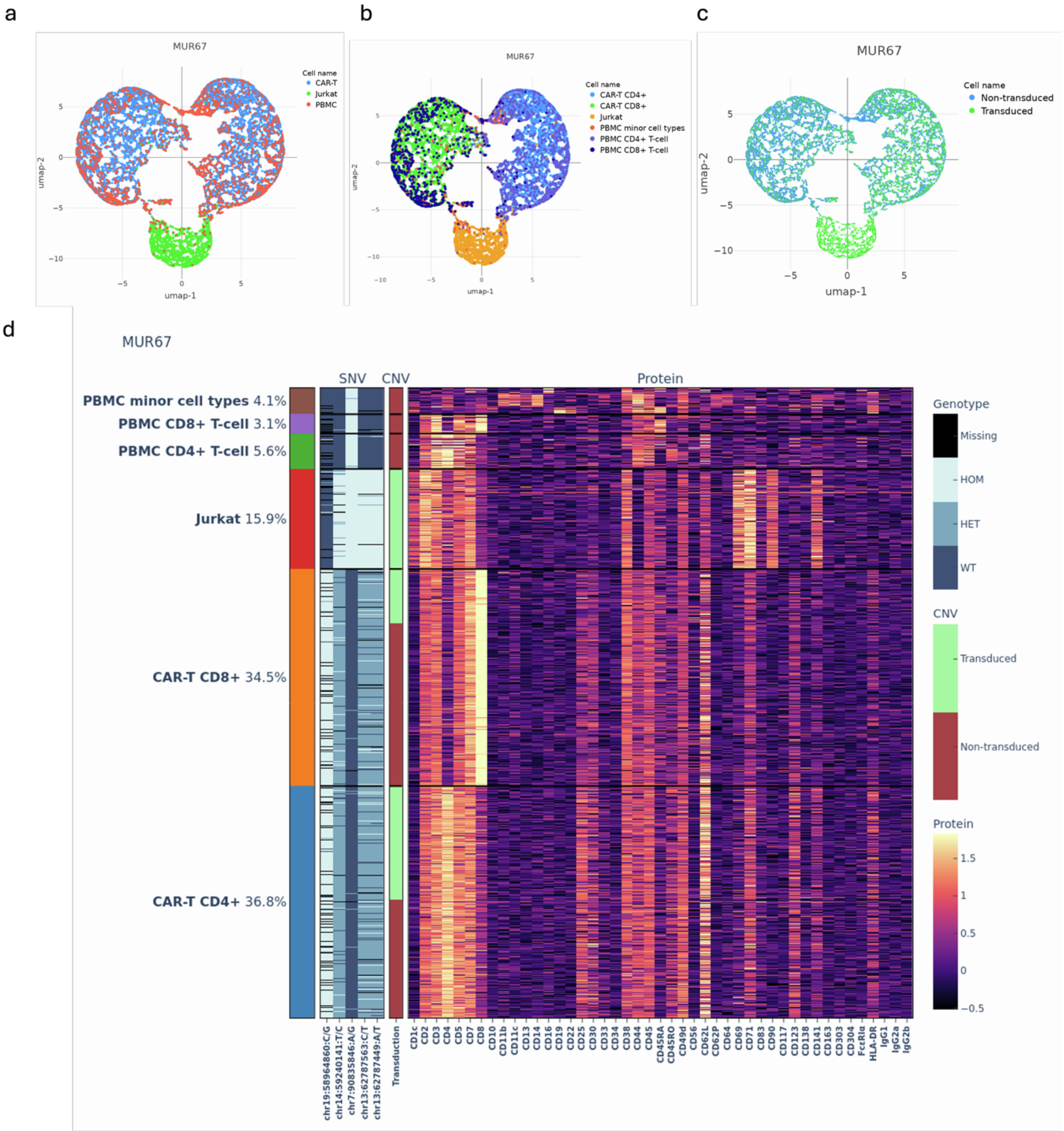

Figure S5. Visualization of cell clustering and genotype, immunophenotyping, and vector transduction status for one of the three replicate Tapestri runs with CAR-T cell product (MUR67). For interpretations please refer to Figure 4. The compositional breakdown of PBMC minor cell types: monocyte: 1.89%, B cell: 1.12%, NK cell: 0.94%, CD69+ T cell: 0.18%.

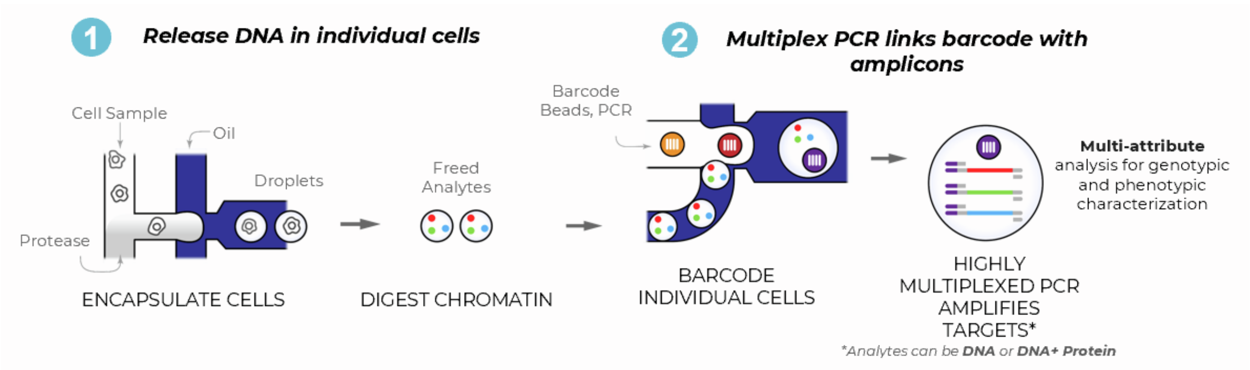

Figure S6 Overview of Mission Bio's Tapestri workflow

Table S1 Amplicon panel used for this study. (Supplemental Table S1.xlsx)

Table S2. Cell line compositions and VCN parameters of Tapestri runs used for this study.

Tapestri runs	Cell line compositions	Control cells	Control VCN	Amplicons
VCN35_V1	V1[a](92%) + RAJI(8%)	Jurkat cells in run VCN39_V4	4	All vector and human amplicons
VCN37_V1	V1(92%) + RAJI(8%)			
VCN42_V1	V1(92%) + RAJI(8%)			
VCN46_V2	V2[b](92%) + RAJI(8%)			
VCN47_V2	V2(92%) + RAJI(8%)			
VCN48_V2	V2(92%) + RAJI(8%)			
VCN49_V3	V3[c](92%) + RAJI(8%)			
VCN50_V3	V3(92%) + RAJI(8%)			
VCN51_V3	V3(92%) + RAJI(8%)			
VCN38_V4	V4[d](92%) + RAJI(8%)			
VCN39_V4	V4(92%) + RAJI(8%)			
VCN40_V4	V4(92%) + RAJI(8%)			

VCN52_Mix1	V1(45%) + V2(25%) + V3(15%) + V4(5%) + RAJI(15%)			
VCN54_Mix1	V1(45%) + V2(25%) + V3(15%) + V4(5%) + RAJI(15%)			
VCN44_Mix2	V1(5%) + V2(15%) + V3(25%) + V4(45%) + RAJI(10%)			
VCN45_Mix2	V1(5%) + V2(15%) + V3(25%) + V4(45%) + RAJI(10%)			
VCN55_Mix3	V1(67%) + V4(23%) + RAJI(10%)			
VCN56_Mix3	V1(67%) + V4(23%) + RAJI(10%)			
VCN57_Mix3	V1(67%) + V4(23%) + RAJI(10%)			
MUR10_MixA	Jurkat VCN 0 (100%)	Jurkat cells from run MUR62	2	Subset of vector and human amplicons[g]
MUR11_MixA	Jurkat VCN 0 (100%)			
MUR12_MixA	Jurkat VCN 0 (100%)			
MUR44_MixC	Jurkat VCN0(47.5%) + CAR Jurkat[e] VCN2(47.5%) + GM12878 VCN 0(5%)			
MUR45_MixC	Jurkat VCN0(47.5%) + CAR Jurkat VCN2(47.5%) + GM12878 VCN 0(5%)			
MUR46_MixC	Jurkat VCN0(47.5%) + CAR Jurkat VCN2(47.5%) + GM12878 VCN 0(5%)			
MUR47_MixD	Jurkat VCN0(23%) + CAR Jurkat VCN2(72%) + GM12878 VCN0(5%)			
MUR48_MixD	Jurkat VCN0(23%) + CAR Jurkat VCN2(72%) + GM12878 VCN0(5%)			
MUR49_MixD	Jurkat VCN0(23%) + CAR Jurkat VCN2(72%) + GM12878 VCN0(5%)			
MUR50_MixE	CAR Jurkat VCN2(95%) + GM12878 VCN0(5%)			
MUR51_MixE	CAR Jurkat VCN2(95%) + GM12878 VCN0(5%)			
MUR52_MixE	CAR Jurkat VCN2(95%) + GM12878 VCN0(5%)			
MUR62	CAR Jurkat VCN 2 (60%) + PBMC (40%)			
MUR65	CAR-T[f] (60%) + CAR Jurkat VCN2 (25%) + PBMC VCN0 (15%)	Spike-in Jurkat VCN2 cells		
MUR66	CAR-T (60%) + CAR Jurkat VCN2 (25%) + PBMC VCN0 (15%)			
MUR67	CAR-T (60%) + CAR Jurkat VCN2 (25%) + PBMC VCN0 (15%)			

For all runs, maximum copy number 9 was allowed. a-d: Jurkat control cell lines with VCN 1-4 provided by NIST, e: Jurkat VCN 2 reference cell line transduced with CD19xCD22 CAR vector provided by CHC, f: CAR-T cell product provided by CHC, g: vector amplicons excluded: "CO546_AMP19", "CO546_AMP7", "CO546_AMP20", human amplicons excluded "VCN_AMP127", "VCNr_AMP26", "VCNr_AMP23", "VCNr_AMP35",

"VCNr_AMP29", "VCNr_AMP37", "VCNr_AMP33", "VCNr_AMP27", "VCN_AMP339" due to their low performance.

Table S3 Sequencing and panel performance metrics of all Tapestri runs used in this study.

(Supplemental Table S3.xlsx)

a: Panel uniformity: This is the percentage of human amplicons that have mean reads to the amplicon above 0.2 * the mean reads per amplicon per cell.

b: Data completeness: This is defined as the percentage of amplicon per cell combinations that have more than 10 reads.

For further information, please refer to Tapestri platform support website:

https://support.missionbio.com/hc/en-us/articles/360053187154-File-report-html#Panel-Uniformity

Table S4. Performance metrics of Tapestri single-cell VCN caller in detecting transduction in NIST control cell lines.

	Transduced	Not transduced
Detected transduction	77,654 cells	25 cells
No transduction detected	288 cells	4,136 cells

Table S5 Single-cell VCN and ddPCR VCN results of all runs tested in this study.

Runs	cn-0	cn-1	cn-2	cn-3	cn-4	cn-5	cn-6	cn-7	cn-8	cn-9	Average VCN	ddPCR VCN[a]
VCN35_V1	0.00	1.00	0.00	0.00	0.00	0.00	0.00	0.00	0.00	0.00	1.00	1.03
VCN37_V1	0.00	1.00	0.00	0.00	0.00	0.00	0.00	0.00	0.00	0.00	1.00	0.98
VCN42_V1	0.00	1.00	0.00	0.00	0.00	0.00	0.00	0.00	0.00	0.00	1.00	0.99
VCN46_V2	0.00	0.07	0.93	0.00	0.00	0.00	0.00	0.00	0.00	0.00	1.93	1.99
VCN47_V2	0.00	0.02	0.98	0.00	0.00	0.00	0.00	0.00	0.00	0.00	1.98	2.08
VCN48_V2	0.00	0.04	0.96	0.00	0.00	0.00	0.00	0.00	0.00	0.00	1.96	1.96
VCN49_V3	0.00	0.00	0.23	0.77	0.00	0.00	0.00	0.00	0.00	0.00	2.77	2.66
VCN50_V3	0.00	0.00	0.03	0.97	0.00	0.00	0.00	0.00	0.00	0.00	2.97	3.00
VCN51_V3	0.00	0.00	0.14	0.86	0.00	0.00	0.00	0.00	0.00	0.00	2.86	2.86
VCN38_V4	0.00	0.00	0.00	0.18	0.82	0.00	0.00	0.00	0.00	0.00	3.82	4.33
VCN39_V4	0.00	0.00	0.00	0.00	0.94	0.06	0.00	0.00	0.00	0.00	4.06	4.33
VCN40_V4	0.00	0.00	0.00	0.01	0.99	0.00	0.00	0.00	0.00	0.00	3.99	4.36
VCN52_Mix1_rep	0.11	0.35	0.22	0.22	0.09	0.01	0.00	0.00	0.00	0.00	1.92	1.64
VCN54_Mix1_PP	0.07	0.44	0.24	0.21	0.02	0.01	0.01	0.01	0.00	0.00	1.75	1.72
VCN44_Mix2_PP	0.07	0.05	0.15	0.31	0.36	0.05	0.00	0.00	0.00	0.00	3.00	2.82
VCN45_Mix2_PP	0.07	0.03	0.12	0.25	0.47	0.06	0.00	0.00	0.00	0.00	3.22	2.93
VCN55_Mix3_PP	0.10	0.66	0.00	0.00	0.23	0.01	0.00	0.00	0.00	0.00	1.64	1.57
VCN56_Mix3_PP	0.09	0.65	0.00	0.03	0.21	0.01	0.00	0.00	0.00	0.00	1.67	1.63
VCN57_Mix3_PP	0.08	0.65	0.00	0.00	0.15	0.10	0.01	0.00	0.00	0.00	1.89	1.63
MUR10_MixA	1.00	0.00	0.00	0.00	0.00	0.00	0.00	0.00	0.00	0.00	0.01	0.00

MUR11_MixA	1.00	0.00	0.00	0.00	0.00	0.00	0.00	0.00	0.00	0.00	0.01	0.00
MUR12_MixA	1.00	0.00	0.00	0.00	0.00	0.00	0.00	0.00	0.00	0.00	0.01	0.00
MUR44_MixC	0.53	0.00	0.43	0.04	0.00	0.00	0.00	0.00	0.00	0.00	1.21	0.88
MUR45_MixC	0.54	0.00	0.46	0.00	0.00	0.00	0.00	0.00	0.00	0.00	0.94	0.88
MUR46_Mixc	0.53	0.00	0.47	0.00	0.00	0.00	0.00	0.00	0.00	0.00	1.08	0.88
MUR47_MixD	0.25	0.00	0.75	0.00	0.00	0.00	0.00	0.00	0.00	0.00	1.49	1.49
MUR48_MixD	0.26	0.04	0.70	0.00	0.00	0.00	0.00	0.00	0.00	0.00	1.45	1.49
MUR49_MixD	0.25	0.00	0.75	0.00	0.00	0.00	0.00	0.00	0.00	0.00	1.45	1.49
MUR50_MixE	0.00	0.00	0.87	0.13	0.00	0.00	0.00	0.00	0.00	0.00	1.97	2.23
MUR51_MixE	0.00	0.00	1.00	0.00	0.00	0.00	0.00	0.00	0.00	0.00	2.13	2.23
MUR52_MixE	0.00	0.00	0.75	0.25	0.00	0.00	0.00	0.00	0.00	0.00	2.11	2.23
MUR65 CAR-T	0.63	0.23	0.05	0.06	0.02	0.01	0.01	0.00	0.00	0.00	0.7	NA
MUR65 CAR-T CD4+	0.51	0.25	0.09	0.07	0.04	0.02	0.01	0.01	0.00	0.00	1.05	NA
MUR65 CAR-T CD8+	0.75	0.19	0.03	0.02	0.00	0.00	0.00	0.00	0.00	0.00	0.34	NA
MUR65 CAR-T	0.64	0.20	0.07	0.03	0.02	0.01	0.01	0.00	0.00	0.00	0.69	NA
MUR65 CAR-T CD4+	0.51	0.24	0.11	0.06	0.03	0.02	0.01	0.01	0.00	0.00	1.06	NA
MUR65 CAR-T CD8+	0.78	0.17	0.03	0.01	0.00	0.00	0.00	0.00	0.00	0.00	0.32	NA
MUR65 CAR-T	0.64	0.18	0.09	0.04	0.02	0.01	0.01	0.00	0.00	0.00	0.72	NA
MUR65 CAR-T CD4+	0.52	0.21	0.12	0.07	0.04	0.02	0.01	0.01	0.00	0.00	1.07	NA
MUR65 CAR-T CD8+	0.77	0.16	0.06	0.01	0.00	0.00	0.00	0.00	0.00	0.00	0.35	NA

Table shows the proportion of cells with VCN from cn-0 to cn-9. For CAR-T cell product samples, details were shown for each subset of CD4+ or CD8+ T cells. Two runs (MUR63 and MUR64) with CAR-transduced primary T cells were shown separately in Table S9.

a: average of two ddPCR probes when available.

Table S6 Normalized Read Count Differences Between Outlier and Expected VCN Populations

(Supplemental Table S6.xlsx)

Outliers include “low_VCN” (VCN calls lower than expected VCN for each run and above zero) and “high_VCN” (VCN calls higher than expected VCN for each run)

Expected VCN shown in table S2 for each run.

“is_low” or “is_high” columns indicate if read counts are significantly lower or higher than the expected VCN population (one tailed *t*-test alpha = 0.001)

Table S7 Vector mutation detected in NIST reference cell lines

Tapestri runs	Total cells	Mutated cells	Mutated cells' mean VCN	Mutated %	Detected VCN4	Intended VCN4
VCN52_Mix1_rep	6708	313	5.20	4.67%	8.97%	5.00%
VCN44_Mix2_PP	3456	1567	4.81	45.34%	36.16%	45.00%
VCN45_Mix2_PP	5960	2802	4.95	47.01%	46.88%	45.00%
VCN55_Mix3_PP	5297	1266	4.64	23.90%	22.79%	23.00%
VCN56_Mix3_PP	3231	769	4.58	23.80%	21.34%	23.00%
VCN57_Mix3_PP	4792	1247	5.09	26.02%	15.39%	23.00%

Table S8. Transduction detection values across admixtures with defined VCN ratios. The table presents the measured transduction detection values for each VCN0:VCN2 ratio, along with the coefficient of variation (CV%) calculated from replicate measurements. Each concentration was tested in three replicates to assess measurement consistency and variability.

Tapestri runs	Cell line compositions	Non-transduced	Transduced	Transduction %	CV %	Tapestri average VCN	Tapestri group average VCN	(dPCR) 41BB	(dPCR) FMC63	ddPCR group average VCN
MUR10_ MixA	VCN 0 (100%)	100%	0%	0	NA	0.01	0.01			0
MUR11_ MixA	VCN 0 (100%)	100%	0%	0		0.01	0.01	0.00	0.00	0
MUR12_ MixA	VCN 0 (100%)	100%	0%	0		0.01	0.01	0.00	0.00	0
MUR44_ MixC	VCN 0, 2 (50%:50%)	50%	50%	45.91%	1.30 %	1.21	1.08	0.91	0.84	0.88
MUR45_ MixC	VCN 0, 2 (50%:50%)	50%	50%	45.19%		0.94	1.08	0.91	0.87	0.88
MUR46_ MixC	VCN 0, 2 (50%:50%)	50%	50%	46.34%		1.08	1.08			0.88
MUR47_ MixD	VCN 0, 2 (25%:75%)	25%	75%	73.48%	0.90 %	1.49	1.47	1.50	1.49	1.49
MUR48_ MixD	VCN 0, 2 (25%:75%)	25%	75%	72.13%		1.45	1.47	1.51	1.45	1.49
MUR49_ MixD	VCN 0, 2 (25%:75%)	25%	75%	72.74%		1.45	1.47			1.49

MUR50_MixE	VCN 2 (100%)	0%	100%	98.83%	0.40 %	1.97	2.07	2.34	2.23	2.23
MUR51_MixE	VCN 2 (100%)	0%	100%	99.53%		2.13	2.07	2.27	2.09	2.23
MUR52_MixE	VCN 2 (100%)	0%	100%	98.94%		2.11	2.07			2.23

Table S9. Performance metrics of Tapestri single-cell VCN caller in detecting transduction in CD19 and CD22 bi-cistronic CAR vector reference cell lines.

Measured\Expected	VCN2 Jurkat Transduced	VCN0 Jurkat Non-transduced	VCN0 GM12878 Non-transduced
Detected transduction	20,093 cells (TP)	0 cells (FP)	5 cells (FP)
No transduction detected	164 cells (FN)	9505 cells (TN)	428 cells (TN)

TP: True positive, FP: False positive, TN: True negative, FN: False negative.

Table S10: Percentage of CAR-transduced primary T-cells with VCN estimated by Tapestri single-cell assay and predicted by Poisson distribution model

VCN	Tapestri replicate 1 (Run ID: MUR63)	Tapestri replicate 2 (Run ID: MUR64)	Poisson model predicted percentage
0	18.82	18.46	19.79
1	35.28	34.86	32.06
2	25.90	21.78	25.97

3	14.12	17.80	14.02
4	3.47	4.70	5.68
5	0.99	1.08	1.84
6	0.56	0.50	0.50
7	0.42	0.35	0.11
8	0.28	0.26	0.02
9	0.16	0.19	0.00

Table S11 Proxy exhaustion-like phenotype detection in effector memory T cells with and without CAR

Sample	CAR status	CD69+ & HLA-DR+ cells	Not (CD69+ & HLA-DR+) cells	Total effector memory T cells	Percentage of double positive cells	Fisher exact odd ratio	Fisher exact p-value
MUR65	CAR+	68	432	500	13.6%	1.45	0.0307
	CAR-	107	984	1091	9.81%		
MUR66	CAR+	16	126	142	11.27%	0.683	0.258
	CAR-	56	301	357	15.69%		
MUR67	CAR+	57	441	498	11.45%	1.34	0.117
	CAR-	92	955	1047	8.79%		
